# Supplementary material for: 3D-CLEM Reveals that a Major Portion of Mitotic Chromosomes Is Not Chromatin
Source: Mol Cell. 2016 Nov 17;64(4):790–802. doi: 10.1016/j.molcel.2016.10.009 (PMC5128728; doi:10.1016/j.molcel.2016.10.009)
Supplement: Document S1. Supplemental Experimental Procedures, Figures S1–S5, and Table S1 [file mmc1.pdf]

Molecular Cell, Volume *64*

Supplemental Information

3D-CLEM Reveals that a Major Portion of Mitotic Chromosomes Is Not Chromatin

Daniel G. Booth, Alison J. Beckett, Oscar Molina, Itaru Samejima, Hiroshi Masumoto, Natalay Kouprina, Vladimir Larionov, Ian A. Prior, and William C. Earnshaw

Supplementary Figure 1 (related to Figure 2)

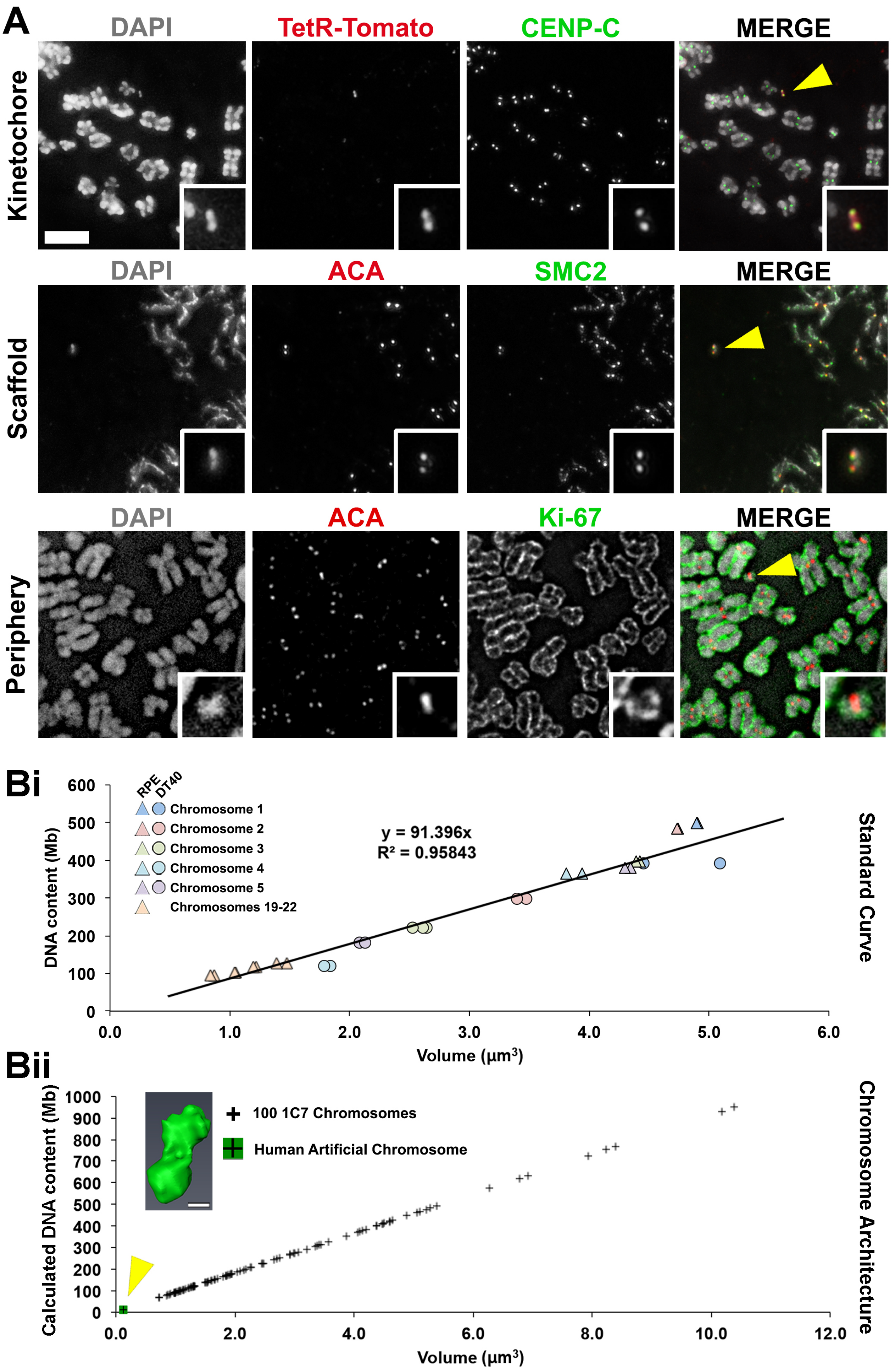

Supplementary Fig. 1: A human artificial chromosome (HAC) contains components from all major chromosome compartments. A) 1C7 cells were used for metaphase spreads. Chromosomes were probed with antibodies targeting major chromosome compartments: The kinetochore (CENP-C), the chromosome scaffold (SMC2) and the chromosome periphery (Ki-67). DAPI, human anti-centromere autoantibodies (ACA) or TetR-tomato were used to identify the HAC. Bi) A scatter plot of volume versus DNA content for chromosomes 1-5 from both RPE (triangles) and DT40 (circles) cells analyzed in Figs. 1 and 4. Line of best fit allows Y value to be determined. Bii) A 2D plot of volume versus DNA content for 1C7 HAC data. Using the standard curve from Suppl Fig. 1Bi, DNA content of all 1C7 chromosomes was estimated, using chromosome volume as the known variable. The DNA content of the HAC (green box and image, inset) was also predicted. Bar = A - 4 µm, H – 250 nm.

Supplementary Figure 2 (related to Figure 4)

A

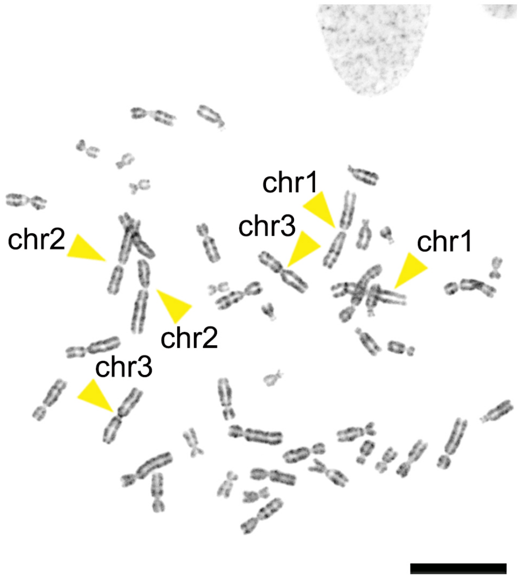

B

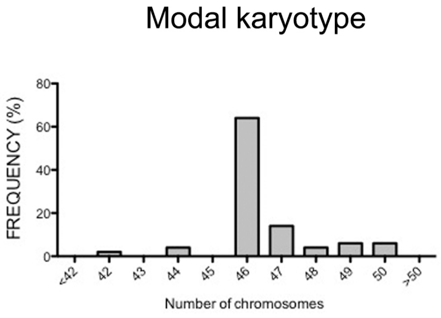

Supplementary Fig. 2: RPE1-hTERT cells maintain a stable modal karyotype of 46 chromosomes. A) Representative RPE1-hTERT metaphase chromosome spread. Chromosomes are stained with DAPI. Yellow arrowheads point to chromosomes 1, 2 and 3 identified by DAPI banding. **B)** Modal karyotype of the RPE1-hTERT cell line. The histogram shows the frequency in which different chromosome numbers were observed in 50 individual metaphases.

Supplementary Figure 3 (related to Figure 3)

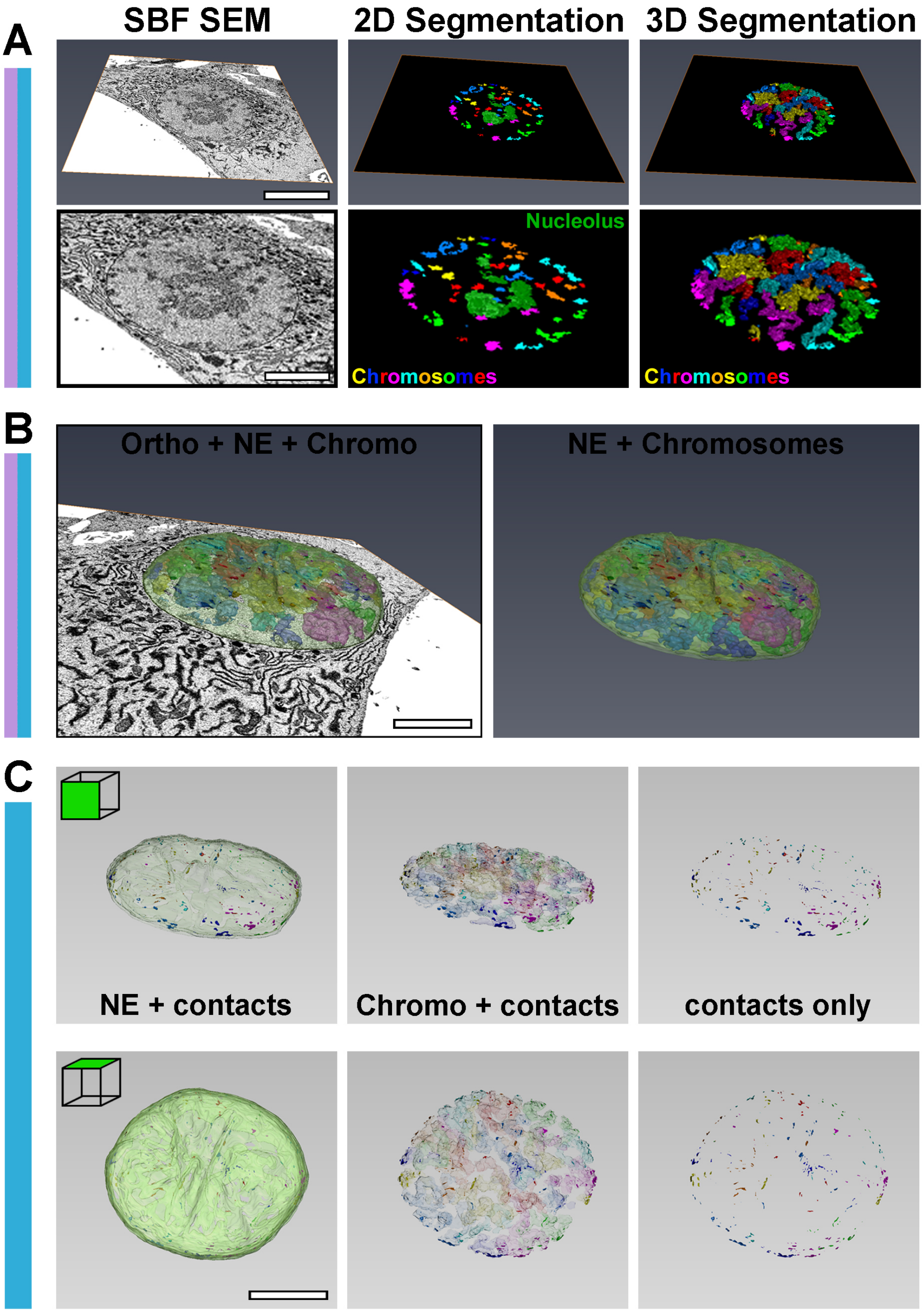

Supplementary Fig. 3: Almost all prophase chromosomes make contact with the NE. A) Representative cross section of a prophase cell. Visualised as EM orthoslice (left panels), 2D segmented chromosome orthoslice (middle panels), segmented chromosomes 2D ortholice plus 3D chromosome model. Bottom panel shows 2X zoom of the top panel. **B)** Reconstruction of the nuclear envelope (NE), shown with and without orthoslice. **C)** Digital footprint of chromosome/NE contact points. Data shown as NE (translucent green) plus contact points (left panels), contact points plus associated chromosome body (central panels), contact points alone (right panels). Chromosome/NE contact points have been left opaque in all images. Bars: A top – 10 µm, bottom – 5 µm; B and C – 5 µm.

Supplementary Figure 4 (related to Figures 3, 4 and 6)

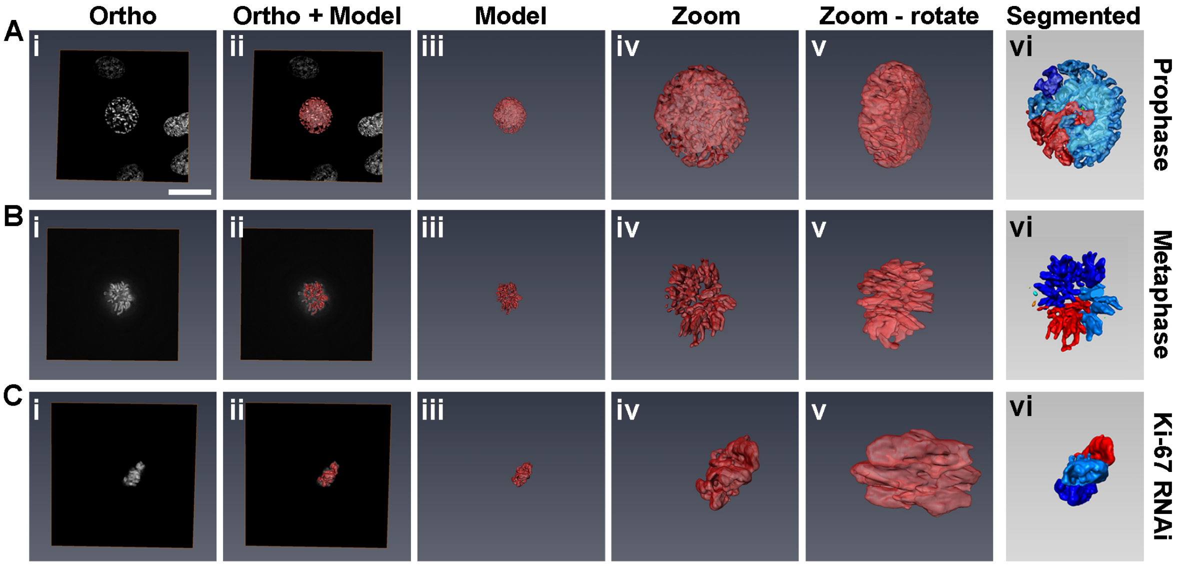

D

		Prophase	Metaphase	Ki67 RNAi
LIGHT MICROSCOPE	Volume (μm^3)	635	256	352
	Surface Area (μm^2)	1970	1196	585
	Chromosomes Segmented	8/46	14/46	4/46
ELECTRON MICROSCOPE	Volume (μm^3)	109.8	175.96	170.23
	Surface Area (μm^2)	1175.73	899.3	986.42
	Chromosomes Segmented	43/46	46/46	20/46

Supplementary Fig. 4: Direct comparison of correlative 3D modeling using DAPI and SBF-SEM reveals large discrepancies. Deconvolved DAPI signals from prophase (A), metaphase (B) and Ki-67 depleted cells (C), were modeled using Amira. Images show orthoslice alone (i), orthoslice plus model (ii) and model alone (iii-v). Segmentation tools, with identical parameters of their SBF-SEM counterparts, were attempted (vi). D) Summary table containing measurements from prophase, metaphase and Ki-67 depleted cells obtained from both light and electron microscopy informed data.

Supplementary Figure 5 (related to Figures 3 and 4)

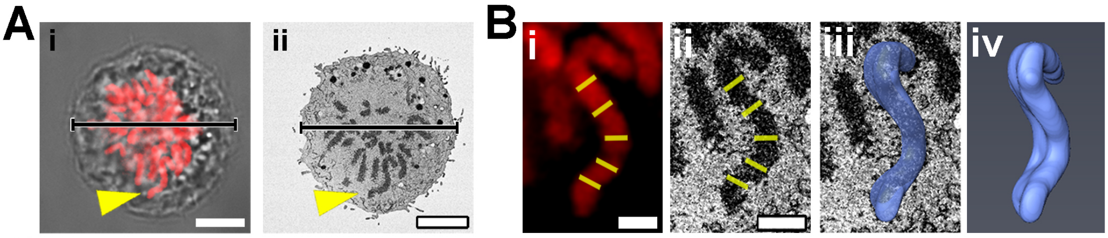

C

CELL		CHROMOSOME	
Light	EM	DAPI	EM
19.57 µm ± 1.03	18.66 µm ± 0.05	0.70 µm ± 0.07	0.69 µm ± 0.01

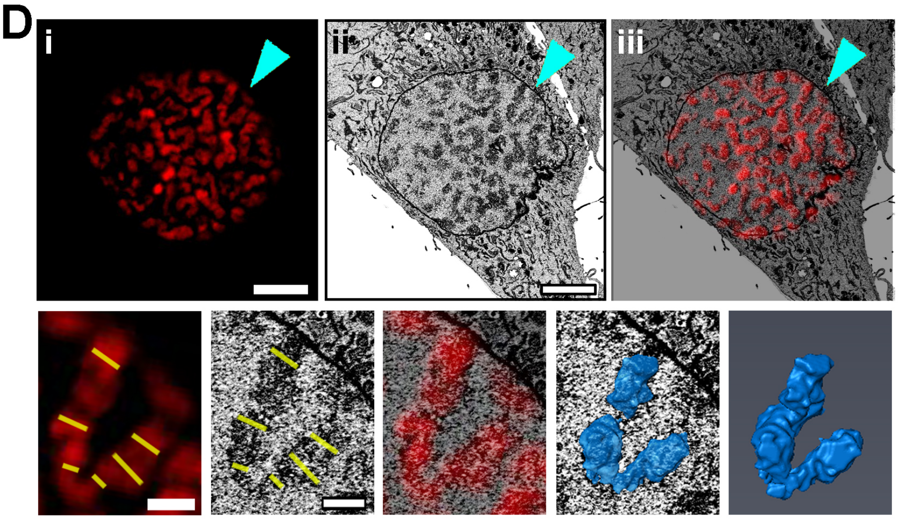

E

CHROMOSOME	
DAPI	EM
0.525 µm ± 0.124	0.528 µm ± 0.129

Supplementary Fig. 5: Direct comparison of 2D data acquired using DAPI and SBF-SEM are identical. Metaphase analysis (A-C). A) Whole cell comparison of metaphase. Metaphase cell diameter was measured using LM and SBF-SEM images. B) Zoom of chromosome from A (yellow arrow). Chromosome diameter was measured from correlative light (i) and SBF-SEM images (ii). The SBF-SEM informed model is also shown (iii-iv). C) Summary table of measurements. Prophase analysis (D-E). D) Correlative optical (i) and physical (ii) sections were used to measure prophase chromosome diameter. Overlay shown in iii. Zoom of chromosome from Di-iii (cyan arrow), represent an example of how chromosomes were measured using correlative light and SBF-SEM images. The SBF-SEM informed model is also shown (blue). E) Summary table of measurements. N = $6_{measurements}$ x $5_{chromosomes}$ for both prophase and metaphase cells. Bars: A, D = 5 µm, B, D (zoom) = 1 µm.

Supplementary Table 1 (related to Figures 4 and 5)

Segmented Units	Predicted Chromosome	Volume (um^3)	Surface Area (um^2)	DNA content (MB)	Compaction (MB per um^3 of chromosome envelope)	Length (um)	Width (um)	Width StDev (um)	Distance from Cell Center (um)	Volume W/O chromosome periphery (um^3)	Compaction (MB per um^3 of chromosome W/O periphery)
1	1	7.09	40.65	498	70.240	8.76	1.14	0.18	5.71	4.89	101.80
2	1	7.07	37.21	498	70.432	8.27	1.00	0.10	4.65	4.88	102.07
3	2	6.85	33.75	484	70.659	8.15	1.14	0.14	6.08	4.73	102.40
4	2	6.84	33.52	484	70.805	7.89	1.16	0.17	6.38	4.72	102.62
5	3	6.39	34.34	396	61.950	7.07	1.25	0.17	6.78	4.41	89.78
6	3	6.34	33.32	396	62.444	7.36	1.29	0.11	6.41	4.38	90.50
7	4	6.28	29.88	380	60.479	7.83	1.19	0.18	6.46	4.34	87.65
8	4	6.21	29.33	380	61.223	6.40	1.16	0.17	5.31	4.28	88.73
9	5	5.70	26.78	364	63.895	6.54	1.12	0.13	7.12	3.93	92.60
10	5	5.50	26.62	364	66.142	6.70	1.19	0.17	7.23	3.80	95.86
39	20	2.13	11.53	128	60.004	3.65	1.01	0.09	3.65	1.47	86.96
40	20	2.01	11.84	128	63.556	3.18	1.21	0.08	3.30	1.39	92.11
41	19	1.76	10.17	118	67.011	3.29	1.06	0.10	3.71	1.22	97.12
42	19	1.73	10.13	118	68.245	3.17	1.15	0.09	3.72	1.19	98.91
43	22	1.51	9.38	102	67.757	2.28	1.21	0.08	1.41	1.04	98.20
44	22	1.50	9.60	102	67.932	1.98	1.16	0.08	3.14	1.04	98.45
45	21	1.26	8.06	94	74.584	2.14	1.15	0.08	2.63	0.87	108.09
46	21	1.21	8.09	94	77.786	1.96	1.13	0.09	3.51	0.83	112.73
**Units 1-10, 39-46**	**SUM**	**77.4 um^3**	**404.2 um^2**	**5128 MB**	**Average of 66.95 Mb per um^3**	**NA**	**1.15 um**	**0.12 um**	**NA**	**53.39**	**Average of 97.03 Mb per um3**
**Units 1 - 46**	**COLLECTIVE SUM**	**175.96 um^3**	**899.3 um^2**	**12344 MB**	**Average of 70.15 Mb per um^3**	**NA**	**NA**	**NA**	**NA**	**121.46**	**Average of 101.55 Mb per um^3**

Supplementary Table 1: Summary table showing chromosome geometry measurements for characterised metaphase chromosomes, and collective summary from all 46 chromosomes (bottom row).

Supplementary Video – Figure Legends

Supplementary Video 1: Video of an alphoid^{tetO} HAC cell showing EM orthoslice (grey scale), modeled chromosomes (blue) and the HAC (green). Suppl. Video 1 related to Figure 2.

Supplementary Video 2: Video of an RPE1-hTERT cell in prophase showing EM orthoslice (grey scale), modeled chromosomes (red), and segmented chromosomes (multi-coloured). NE is shown in green. Digital footprint of chromosome to NE contacts is also shown. Suppl. Video 2 related to Figure 3.

Supplementary Video 3: Video of an RPE1-hTERT cell in metaphase showing EM orthoslice (grey scale), modeled chromosomes (red), and segmented chromosomes (multi-coloured). Suppl. Video 3 related to Figure 4.

SUPPLEMENTARY PROCEEDURES

Microscopy of Metaphase Spreads

Cells were treated with 0.2 µg/ml of colcemid for 3 hours before harvesting. Mitotic chromosomes were collected by mitotic shake off and cytospun onto poly-L-lysine coated slides. Samples were immersed in KCM buffer (10 mM Tris pH=8, 120 mM KCl, 20 mM NaCl, 0.5 mM EDTA, 0.1% Triton X-100) and IF staining was performed using KCM buffer with 1%BSA (Invitrogen). Afterwards, cells were fixed with 4% formaldehyde in KCM buffer and DNA was counterstained with DAPI. The following antibodies were used: rabbit anti-CENP-C (R555; 1/500), rabbit anti-SMC2 (1/1000), rabbit anti-Ki-67 (BD Transduction Lab 1/100 (Booth et al., 2014)), human anti-ACA (Earnshaw and Rothfield, 1985) (1/200). Images were acquired using a cooled CCD camera (CH350; Photometrics) on a wide-field microscope (DeltaVision Spectris; Applied Precision) with an 100X NA 1.4 Plan Apochromat lens. The data sets were deconvolved with softWoRx (Applied Precision).

Mass Spectrometry analysis

Correlation analysis data was mined from our proteomics archives (Ohta et al., 2010, Samejima et al., 2015). Protein-pairs with a strong correlation coefficient ($R \geq 0.80$) were extracted and used to draw the network diagram by Cytoscape.

Composite of chromosomes and breakdown of peripheral fractions.

The mass of proteins were deduced from copy number and their molecular weight. Copy number of each protein on DT40 mitotic chromosomes was inferred by intensity based absolute quantification (iBAQ) algorithm. Spectral intensities of MS1 peak were analysed by MaxQuant 1.5.1.2 (Schwanhausser et al., 2011).

Calculations for chromosome geometry referred to within main text:

-1- A priori prediction of thickness of chromosome periphery in mitotic chromosomes.

Consider chromosome 1

Volume = 7.09 μm^3

diameter = 1.14 μm (radius = 0.57 μm)

Calculate the length (h) of chromosome 1, modeling the chromosome as a cylinder:

volume of a cylinder: $V = \pi \times r^2 \times h$

$7.09\ \mu m^3 = \pi \times (0.57\ \mu m)^2 \times h$

$h = 7/(\pi \times 0.3249) = 6.86\ \mu m$

Estimate the DNA volume in chromosome 1:

Total volume of metaphase chromosomes = 175 μm^3

Assumption: volume of prophase chromosomes (110 μm^3) = volume of the chromatin, as the nucleolus has not yet disassembled.

Periphery volume = 175 - 110 μm^3 = 65 μm^3

Fraction of chromosome volume that is chromatin = 110/(110 + 65) = 0.62857

Volume of chromatin in chromosome 1 = 0.62857 x 7.09 μm^3 = 4.457 μm^3

Diameter of chromatin cylinder in chromosome 1:

$V = \pi \times r^2 \times h$

so $4.457\ \mu m^3 = \pi \times r^2 \times 6.86$

$r^2 = 4.457/(\pi \times 6.86) = 0.207$

$r = 0.455$

Diameter of the chromatin = 2r = 0.91

Thickness of periphery of chromosome 1 =

(chromosome diameter – chromatin diameter)/2

= (1.14 - 0.91)/2 = 115 nm

This "first principles" calculation of the periphery thickness gives a value dead in the middle of our observed range of 87 - 150 nm.

However:

-1- This calculation assumes that the compaction of the chromatin is the same in prophase and mitosis. It also ignores the actual thickness of the chromosome periphery measured on mitotic chromosomes.

Measurements of the thickness of the metaphase periphery by EM:

Booth (Booth et al., 2014) ~150 nm

Hernandez-Verdun and co-workers ~ 143 nm (measured by us from their published micrographs (Gautier et al., 1992a).

Assume a thickness of the periphery at metaphase of 150 nm.

If the diameter of chromosome 1 is: 1.14 μm

The diameter of the chromatin cylinder = 1.14 - (2 x 0.15) μm = 0.84 μm

This gives r = 0.42 μm —> this is the radius of the chromatin in chromosome 1

$V_{chromatin} = \pi \times r^2 \times h = \pi \times (0.42\ \mu m)^2 \times 6.86\ \mu m = 3.80\ \mu m^3$

The fraction of the volume of chromosome 1 that is chromatin = 3.80/7.09 = 0.536

If the thickness of the periphery is 150 nm, then 54% of the total metaphase chromosome volume is chromatin.

-3- How much do chromosomes compact between prophase and metaphase?

The total volume of metaphase mitotic chromosomes is 175 μm^3.

0.54 x 175 μm^3 = 93.8 μm^3.

This means that between the prophase cell that we imaged and the metaphase cell that we imaged, the chromatin volume compacted by (110/93.8)-1 x 100 = 17%

-4- Calculation of the predicted periphery thickness in Ki-67-depleted chromosomes:

Total volume of the chromatin in metaphase chromosomes estimated from light microscopy: 256 μm^3.

Total volume of the chromatin in Ki-67-depleted metaphase chromosomes estimated from light microscopy: 352 μm^3.

Ratio of Ki-67/wild-type volume: 1.375

Metaphase chromosome 1 chromatin volume calculated above from EM: 3.80 μm^3.

Normalized volume of Ki-67-depleted metaphase chromosome 1 chromatin: 5.225 μm^3.

Measured total volume of metaphase chromosome 1: 7.09 μm^3.

Measured total volume of wild type chromosomes: 175 μm^3.

Measured total volume of Ki-67-depleted chromosomes: 170 μm^3.

Corrected volume of chromosome (170/175) x 7.09 = 6.89 μm^3.

Since the total volume is so similar, **<u>assume</u>** that the linear measurements (e.g. radius and length) of Ki-67-depleted chromosomes will be similar to wild type:

$V_{chromatin} = \pi \times r^2 \times h$

$5.225\ \mu m^3 = \pi \times r^2 \times 6.86\ \mu m$

$5.225/(\pi \times 6.86)\ \mu m^2 = r^2$

$.4925\ \mu m = r$

$D = 0.985\ \mu m$

Likely thickness of periphery compartment in Ki-67-depleted chromosome 1:

$(1.14 – 0.985)/2 = 77\ \mu m$

Percentage of chromosome 1 that is chromatin = $5.225\ \mu m^3 / 6.89\ \mu m^3 = 76\%$
