# Supplementary figures and images for: 3D-CLEM Reveals that a Major Portion of Mitotic Chromosomes Is Not Chromatin
Source: Mol Cell. 2016 Nov 17;64(4):790–802. doi: 10.1016/j.molcel.2016.10.009 (PMC5128728; doi:10.1016/j.molcel.2016.10.009)

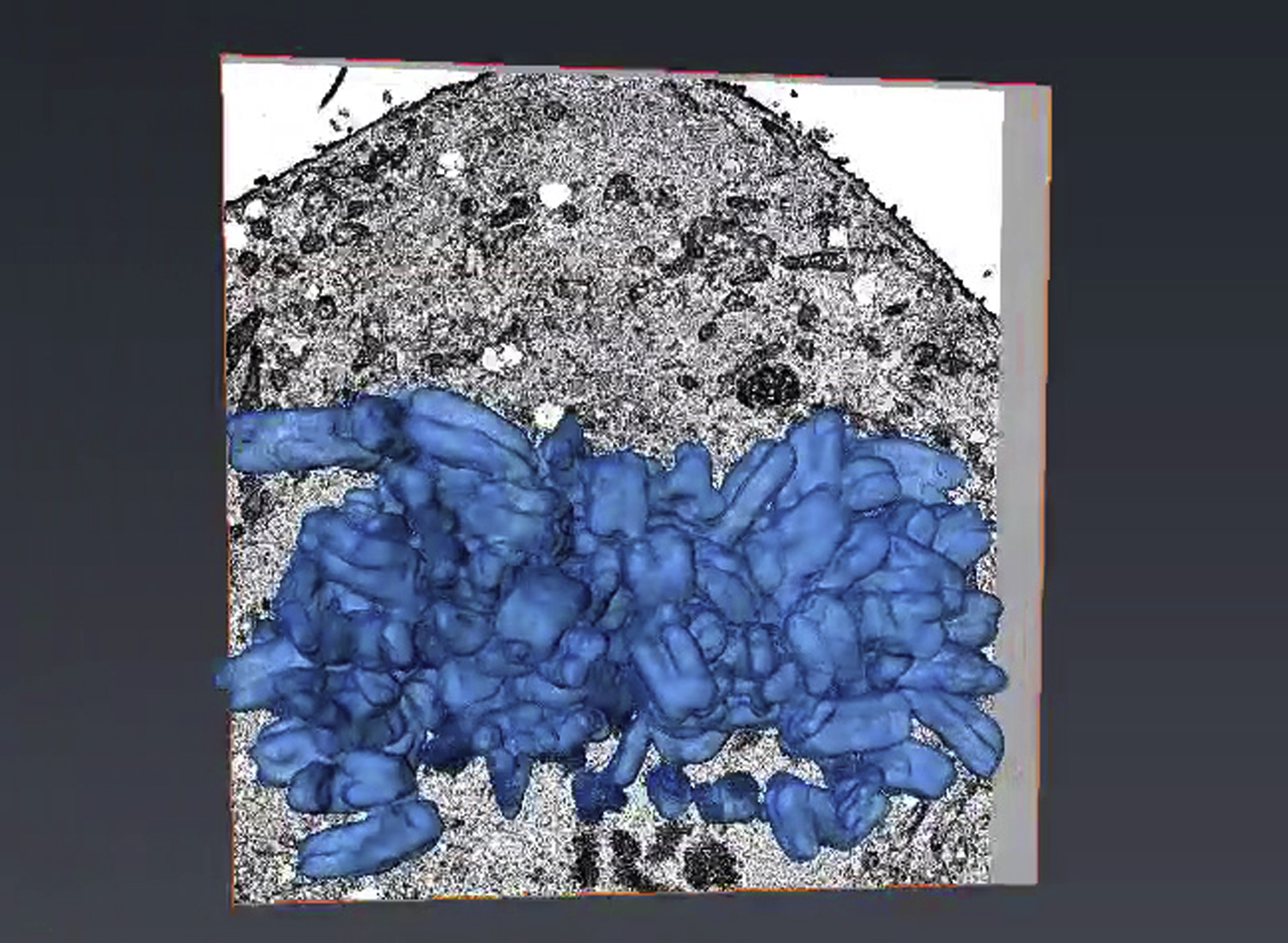

Supplement: Movie S1. AlphoidtetO HAC Cell Showing EM Orthoslice, Modeled Chromosomes, and the HAC, Related to Figure 2 [file mmc2.jpg]

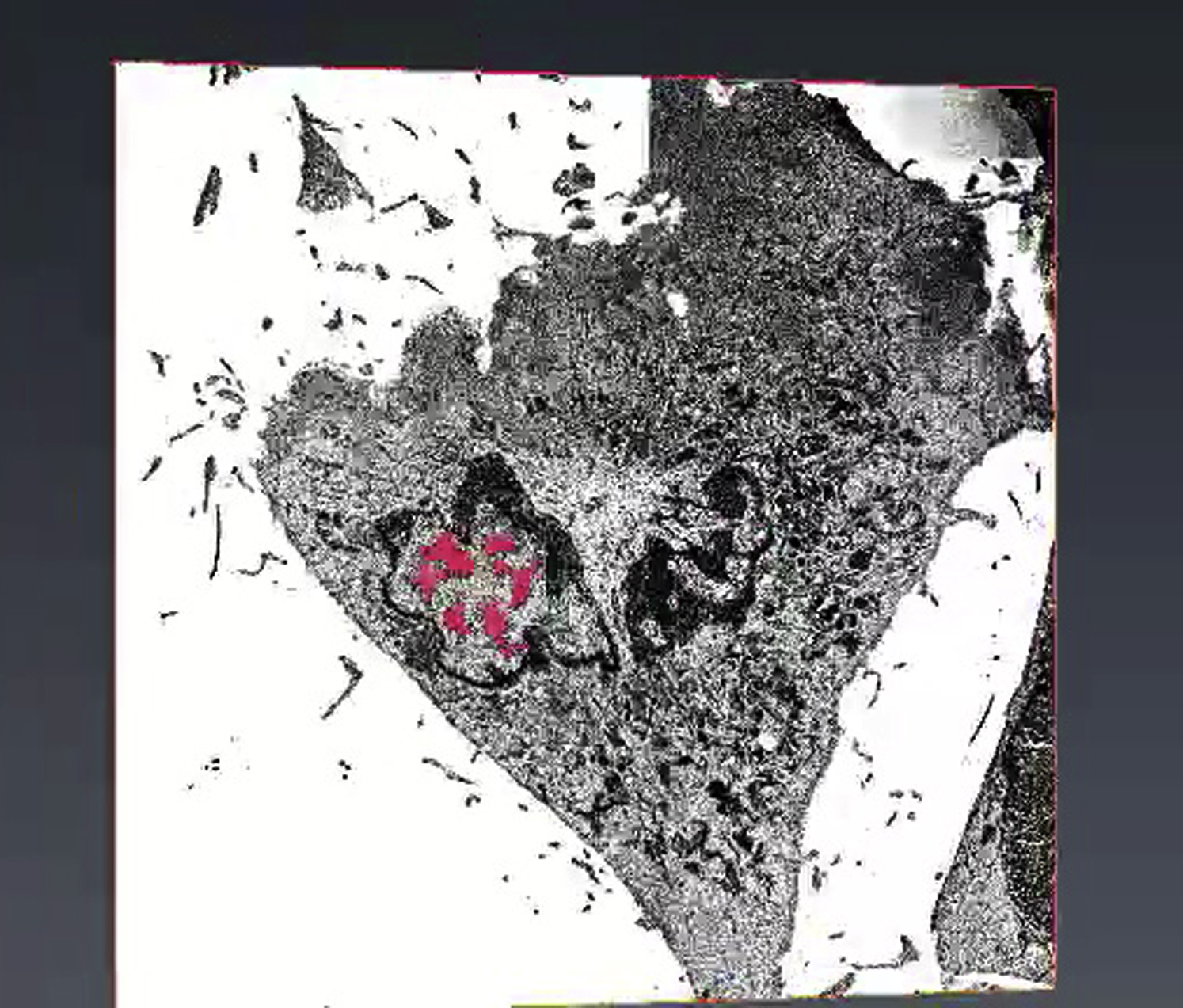

Supplement: Movie S2. RPE1-hTERT Cell in Prophase Showing EM Orthoslice, Modeled Chromosomes, and Segmented Chromosomes — Nuclear envelope (NE) is shown in green. Digital footprint of chromosome to NE contacts is also shown. Movie is related to Figure 3. [file mmc3.jpg]

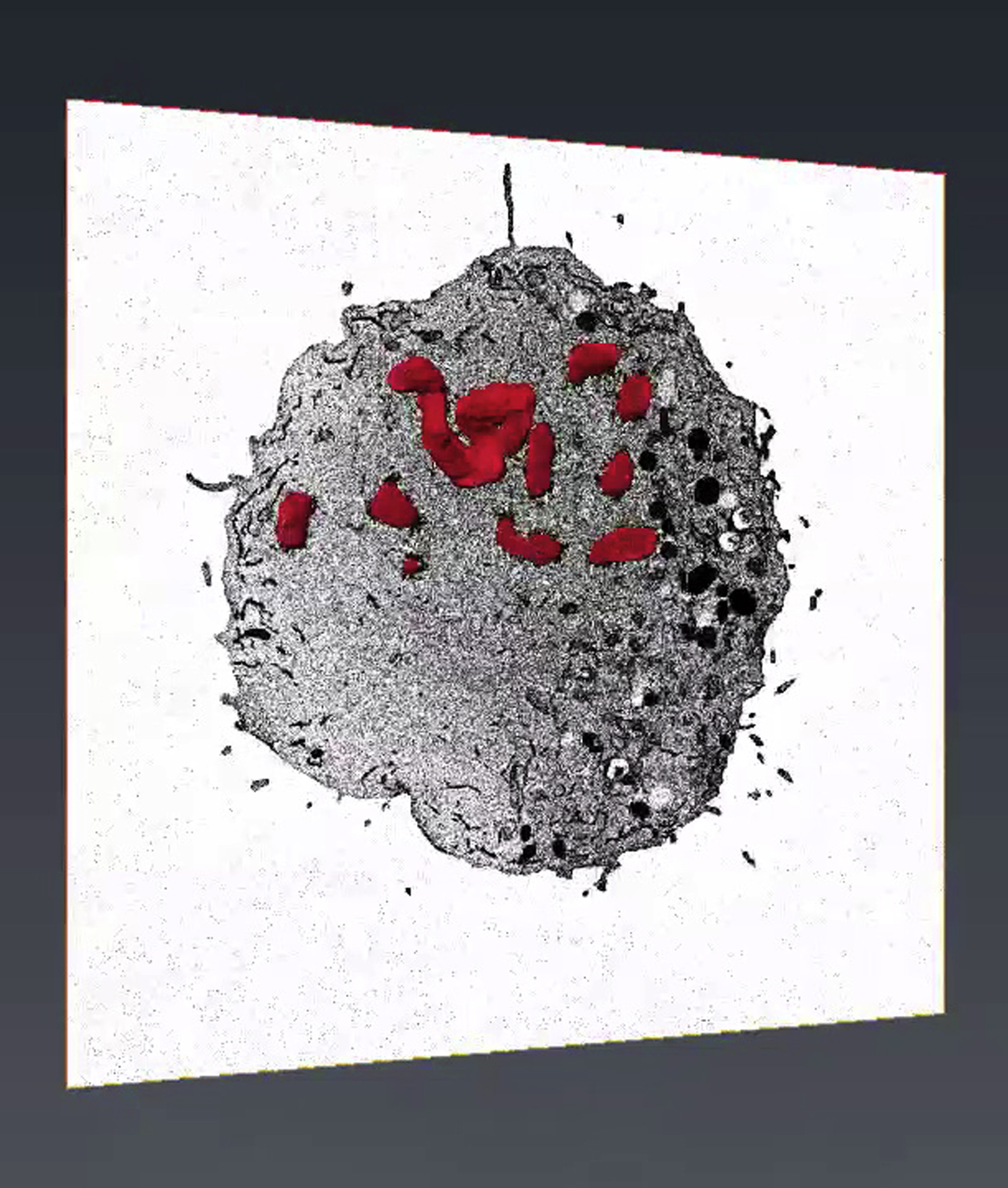

Supplement: Movie S3. RPE1-hTERT Cell in Metaphase Showing EM Orthoslice, Modeled Chromosomes, and Segmented Chromosomes, Related to Figure 4 [file mmc4.jpg]
