# Supplementary material for: 3D-CLEM Reveals that a Major Portion of Mitotic Chromosomes Is Not Chromatin
Source: Mol Cell. 2016 Nov 17;64(4):790–802. doi: 10.1016/j.molcel.2016.10.009 (PMC5128728; doi:10.1016/j.molcel.2016.10.009)
Supplement: Document S2. Article plus Supplemental Information [file mmc5.pdf]

Molecular Cell

Article

3D-CLEM Reveals that a Major Portion of Mitotic Chromosomes Is Not Chromatin

Graphical Abstract

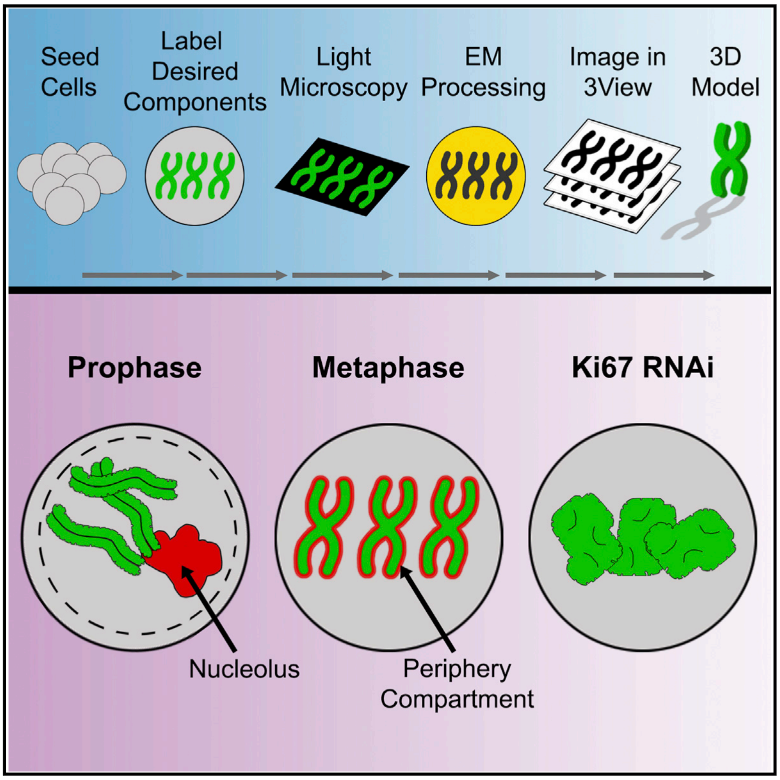

Highlights

- 3D-CLEM combines light and serial block-face scanning electron microscopy
- The complete architecture of all 46 human chromosomes has been defined
- A large portion of mitotic chromosomes is not composed of chromatin
- Chromosome volumes determined by light and electron microscopy differ dramatically

Authors

Daniel G. Booth, Alison J. Beckett, Oscar Molina, ..., Vladimir Larionov, Ian A. Prior, William C. Earnshaw

Correspondence

daniel.booth@ed.ac.uk (D.G.B.),
bill.earnshaw@ed.ac.uk (W.C.E.)

In Brief

Booth et al. develop a microscopy method called 3D-CLEM. Using this approach, they find that a large portion of mitotic chromosomes is not composed of chromatin, challenging classical perceptions of chromosomes.

CrossMark
Booth et al., 2016, Molecular Cell *64*, 790–802
November 17, 2016 © 2016 The Author(s). Published by Elsevier Inc.
http://dx.doi.org/10.1016/j.molcel.2016.10.009

CellPress

3D-CLEM Reveals that a Major Portion of Mitotic Chromosomes Is Not Chromatin

Daniel G. Booth,[1,5,*] Alison J. Beckett,[4] Oscar Molina,[1] Itaru Samejima,[1] Hiroshi Masumoto,[2] Natalay Kouprina,[3] Vladimir Larionov,[3] Ian A. Prior,[4] and William C. Earnshaw[1,*]
[1]Wellcome Trust Centre for Cell Biology, Institute of Cell Biology, University of Edinburgh, EH9 3BF Edinburgh, UK
[2]Department of Frontier Research, Laboratory of Cell Engineering, Kazusa DNA Research Institute, Kisarazu, 292-0818 Chiba, Japan
[3]Developmental Therapeutics Branch, National Cancer Institute, National Institutes of Health, Bethesda, MD 20892-4264, USA
[4]Biomedical Electron Microscopy Unit, Division of Cellular and Molecular Physiology, Institute of Translational Medicine, University of Liverpool, Crown Street, L69 3BX Liverpool, UK
[5]Lead Contact
*Correspondence: daniel.booth@ed.ac.uk (D.G.B.), bill.earnshaw@ed.ac.uk (W.C.E.)
http://dx.doi.org/10.1016/j.molcel.2016.10.009

SUMMARY

Recent studies have revealed the importance of Ki-67 and the chromosome periphery in chromosome structure and segregation, but little is known about this elusive chromosome compartment. Here we used correlative light and serial block-face scanning electron microscopy, which we term 3D-CLEM, to model the entire mitotic chromosome complement at ultra-structural resolution. Prophase chromosomes exhibit a highly irregular surface appearance with a volume smaller than metaphase chromosomes. This may be because of the absence of the periphery, which associates with chromosomes only after nucleolar disassembly later in prophase. Indeed, the nucleolar volume almost entirely accounts for the extra volume found in metaphase chromosomes. Analysis of wild-type and Ki-67-depleted chromosomes reveals that the periphery comprises 30%–47% of the entire chromosome volume and more than 33% of the protein mass of isolated mitotic chromosomes determined by quantitative proteomics. Thus, chromatin makes up a surprisingly small percentage of the total mass of metaphase chromosomes.

INTRODUCTION

Since their first discovery in 1882 (Flemming, 1882), mitotic chromosomes have been a subject of intense study. Remarkably, despite the significant developments of light (LM) and electron microscopy (EM) over the intervening years, the detailed organization of mitotic chromosomes remains a mystery (Belmont, 2002, 2006; Swedlow and Hirano, 2003; Marko, 2008; Kschonsak and Haering, 2015).

Over the years studies of chromosome structure have focused almost entirely on the chromatin. Major controversies have been concerned with the folding of the chromatin fiber, which was first proposed to undergo random spaghetti-like packing (DuPraw, 1966) and more recently envisioned as a polymer melt (Eltsov et al., 2008; Nishino et al., 2012). Others suggested that chromosomes have an organized hierarchy of packing interactions, from helical coiling around the nucleosome (Luger et al., 1997) to a solenoidal 30 nm fiber (Finch and Klug, 1976) to larger gyres (Bajer, 1959; Belmont et al., 1987; Boy de la Tour and Laemmli, 1988; Kireeva et al., 2004; Kuwada, 1939; Ohnuki, 1968; Rattner and Lin, 1985; Sedat and Manuelidis, 1978; Strukov et al., 2003). A third class of models proposes that mitotic chromosomes consist of chromatin loops constrained by non-histone proteins (Adolphs et al., 1977; Paulson and Laemmli, 1977; Marsden and Laemmli, 1979; Earnshaw and Laemmli, 1983). Recent support for this loop model comes from Hi-C studies suggesting that human mitotic chromosomes are composed of a linear array of chromatin loops 80–120 kb in length (Naumova et al., 2013) and from advanced light microscopy methods (Liang et al., 2015).

These models all neglected a thin surface layer that has been recognized on chromosomes by classical light microscopy (Ohnuki, 1968), fluorescence microscopy (Chaly et al., 1984; McKeon et al., 1984), and electron microscopy (Booth et al., 2014; Gautier et al., 1992a). Over the years the periphery was little studied, and its composition was largely undefined (for review, see Van Hooser et al., 2005). Methods for its functional analysis were lacking, and it appeared to be simply a thin, amorphous layer sticking to the chromosome. We recently showed that the chromosome periphery compartment requires the presence of Ki-67 for its assembly (Booth et al., 2014). Ki-67 was discovered as the target of a monoclonal antibody against the nuclei of Hodgkin's lymphoma cells that is now one of the most widely used histological markers for cell proliferation (Whitfield et al., 2006). The gene encodes a huge protein of 3,256 aa that contains 16 repeats of unknown function and that binds chromatin and heterochromatin protein HP1 (Scholzen et al., 2002). Ki-67 is located in the nucleolus during interphase and at the mitotic chromosome periphery from late prophase through telophase of mitosis (Gautier et al., 1992b; Hernandez-Verdun and Gautier, 1994). It has recently been proposed that Ki-67 coats the chromosome surface as a coat of ~80-nm-long "brush-like" structures that functions as a biological surfactant

 © 2016 The Author(s). Published by Elsevier Inc.
This is an open access article under the CC BY license (http://creativecommons.org/licenses/by/4.0/).

(Cuylen et al., 2016). In its absence, chromosomes clump together and nucleolar reactivation is impeded (Booth et al., 2014; Cuylen et al., 2016).

Here we have combined light microscopy, serial block-face scanning electron microscopy (SBF SEM), and modeling to develop a semi-automated data processing pipeline that we term 3D-CLEM. 3D-CLEM has allowed us to determine the length, width, surface area, volume, and DNA packing density of all normal human chromosomes and to determine the DNA content of a small synthetic artificial chromosome (Nakano et al., 2008). Detailed comparison of prophase and metaphase chromosomes (the latter plus and minus Ki-67) yielded several major surprises. First, we were surprised to find that methods used to calculate the volumes of chromosomes based on light microscopy give reproducible, but wildly inaccurate, values. Second, careful analysis of the relative chromosome, chromatin, and periphery volume has revealed that the periphery constitutes a very much larger percentage of the chromosomal volume than recently envisioned. Indeed, in metaphase RPE1 cells, chromatin may comprise as little as 53% of the total chromosome volume. This conclusion that the periphery compartment is much more significant than previously appreciated arose initially from analysis of correlative light and electron microscopy images, but quantitative proteomics analysis also confirmed that putative periphery components comprise more than 33% of the mass of chromosomal proteins. These results reveal that chromosomes are not simply chromatin structures. As a result, earlier physical and structural studies of mitotic chromosomes may need to be reassessed.

RESULTS AND DISCUSSION

Establishing 3D-CLEM: Multimodal Microscopy for Targeted Organelle Analysis

Many key structural features of mitotic chromosomes fall in between the optimal working resolutions of the light and electron microscope. We have therefore developed a pipeline for the three-dimensional analysis of chromosomes by correlative light and serial block-face scanning electron microscopy. This pipeline, which we refer to as 3D-CLEM, allows the modeling of any aspect of chromosome architecture that can be contrasted with electron-dense stains at a resolution of 12–24 × 12–24 × 60 nm, in x, y, and z, respectively. When combined with high-resolution light microscopy, in which individual chromosomal components are imaged using fluorescent labels, this allows a much more comprehensive visualization of the chromosome, or of any other subcellular organelle for which three-dimensional data are desired. The 3D-CLEM pipeline is described in Figure 1.

As a first test of the 3D-CLEM method, we performed a targeted analysis of a synthetic human artificial chromosome (HAC) (Figure 2). HACs are autonomous DNA elements that replicate and segregate accurately during each cell division with an efficiency approaching that of natural chromosomes. HACs have been proposed as potential vectors for gene therapy because they overcome several limitations of current gene delivery methods (Kouprina et al., 2014).

The alphoid^{tetO} HAC was assembled from a circular input DNA construct of 50 kb based on a repeating alpha-satellite dimer having one monomer from chromosome 17 with a CENP-B binding site and one synthetic monomer based on the Choo consensus sequence (Choo et al., 1991) and containing a tetracycline operator in place of the CENP-B box. When introduced into HT1080 fibrosarcoma cells, this 50 kb construct assembled into a fully functional, stable chromosome (Nakano et al., 2008). Previous structural characterization of the synthetic alphoid^{tetO} HAC consisted of molecular analysis of its DNA organization by transformation-associated recombination (TAR) cloning, pulsed field gel electrophoresis, and fiber-FISH (fluorescence in situ hybridization) (Kouprina et al., 2014). During its formation, the HAC acquired 4.2 Mb of DNA from a gene-poor region of the arm of chromosome 13 containing the 407 kb KLHL1 gene (MIM 605332) and the 32 kb ATXN80 gene (MIM 603680), and the alphoid^{tetO} array was expanded to roughly 1.1 Mb (Kouprina et al., 2012, 2014).

We used light microscopy to confirm that the HAC possesses all major chromosome functional compartments. We stained metaphase chromosomes from 1C7 cells, containing a single copy of the alphoid^{tetO} HAC, with antibodies recognizing different chromosome compartments. We could clearly see CENP-C (kinetochore), SMC2 (chromosome scaffold), and Ki-67 (chromosome periphery) on the HAC (Figure S1A). Together, these data suggest that the alphoid^{tetO} HAC is a normal chromosome.

We next analyzed a mitotic 1C7 cell using 3D-CLEM. The alphoid^{tetO} HAC was detected by expressing a tetR-EYFP fusion protein in 1C7 cells. A suitable mitotic cell with the HAC slightly separated from the other chromosomes was identified by LM (Figure 2A), then processed and imaged by SBF SEM. All of the mitotic chromosomes were found in ~600 3View sections (Figure 2B). CLEM registration, using both LM and SBF SEM images, revealed the presence of the HAC in 16 consecutive SBF SEM sections (Figure 2C). When the 2D EM data were converted into a volume format, using ImageJ *volume viewer* (Figures 2D and 2E), we could clearly observe the presence of a normal trilaminar kinetochore on the surface of the alphoid^{tetO} HAC, which had two clearly distinct sister chromatids (Figure 2Eiii, white arrow).

Semi-automated rendering and segmentation identified 101 individual chromosomes in this 1C7 cell (Figures 2Fi–2Fiv; Movie S1). This chromosome number reflects the fact that 1C7 cells are a fusion between HT1080 and HeLa cells (Cardinale et al., 2009). The alphoid^{tetO} HAC was by far the smallest chromosome (volume: 0.122 μm^3, surface area: 2.7 μm^2). Determination of identity of the other chromosomes was not possible due to the large number of chromosomes and structural chromosome reorganizations that occurred during the history of 1C7 and its parental cell lines.

3D-CLEM of Prophase Chromosomes

Imaging of early prophase chromosomes allowed us to observe the condensed mitotic chromatin before it acquired a periphery compartment after nucleolar disassembly.

Using the RPE1 cell line, which has a stable modal karyotype of 46 chromosomes (Figure S2), we identified an early prophase cell using light microscopy (Figure 3Ai) and processed it for SBF SEM (Figure 3Aii). Although prophase chromosomes generate

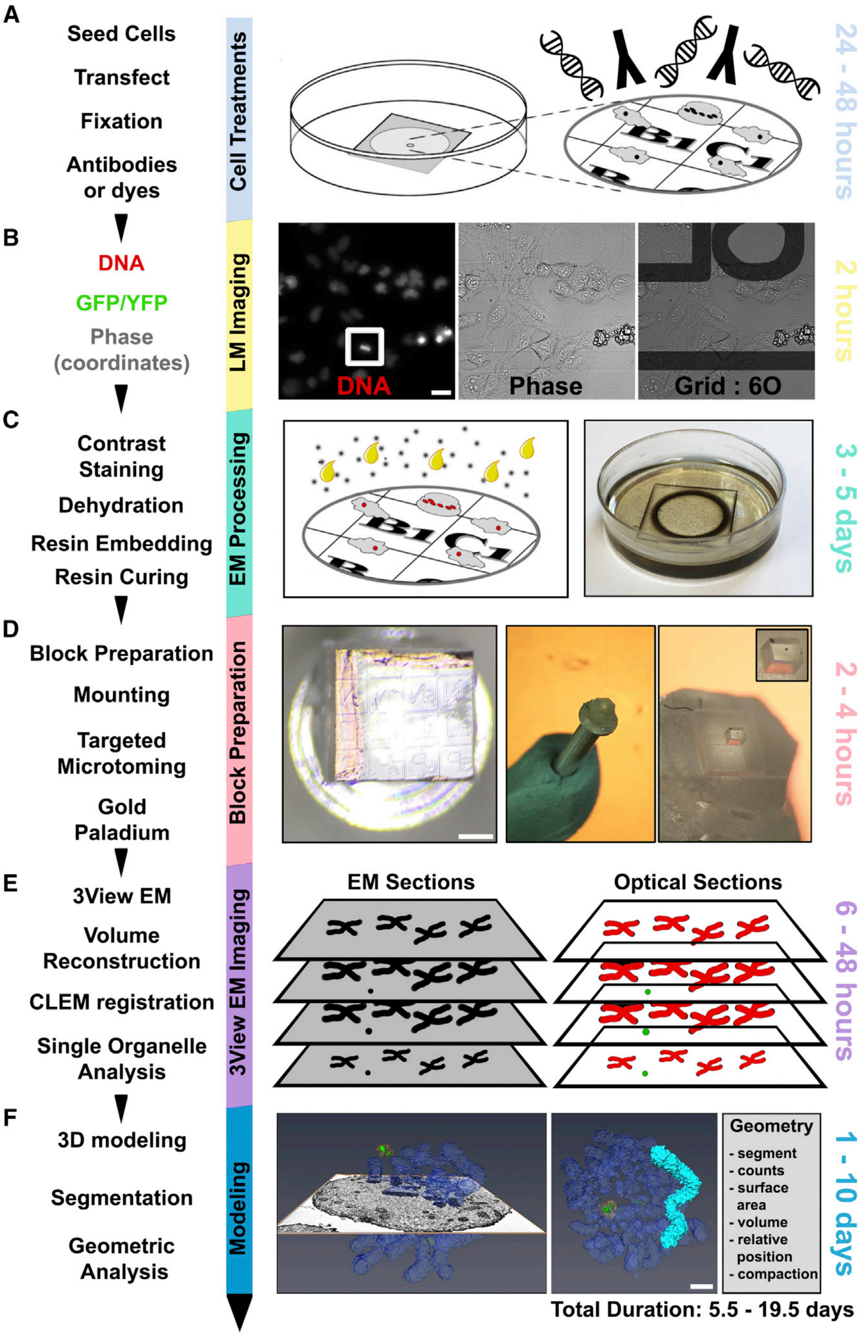

Figure 1. 3D-CLEM Workflow

(A) Cells seeded onto gridded dishes (MatTek) are transfected to express fluorescent fusion proteins. Cells are imaged live or fixed for 1 hr with glutaraldehyde before being treated with dyes (for example, DAPI, to visualize DNA) or probed with antibodies as appropriate (Booth et al., 2011).

(B) Both overview (20×) and high-magnification (100×) light microscopy (LM) images are acquired for the cells of interest. Coverslip coordinates were recorded using phase contrast, aiding location of target cells during later stages of the method.

(C) Samples were stained with osmium, tannic acid, uranyl acetate, and lead aspartate to generate contrast for electron microscopy, then dehydrated with a graded ethanol series before infiltration with resin. Samples were covered in 100% resin and cured at 60°C for 48 hr.

(D) The sample was separated from the dish (Booth et al., 2013), excess resin excised, and the remaining 1 × 1 mm block glued to a pin (left and central panels). Using the coordinates imprinted on the block face, the area of resin containing the cell of interest was fine-trimmed into a 100 × 100 μm block using an ultra-microtome (right panel) and coated with silver paint and gold palladium. A single cell can be observed (far right image).

(E) The sample was mounted into a Gatan 3View microtome, and the block face was repeatedly imaged during the removal of consecutive sections. This provides lossless acquisition in which the entire cell can be imaged and reconstructed. CLEM registration, merging both LM and EM data, was used to identify cells and structures of interest.

(F) Cells and structures of interest were annotated and segmented using Amira software (FEI), resulting in a nanometer resolution, three-dimensional model suitable for further geometric analysis. For preliminary tests, a DT40 chicken lymphoma cell was analyzed and modeled. From start to finish, this method requires ≥5.5 days, subject to the time dedicated to image annotation and data analysis. The typical resolution of the generated models is 12–24 × 12–24 × 60 nm in x, y, and z, respectively (60 nm was the thickness of the sections cut in the 3View).

Scale bars, 20 μm (B); 200 μm (D); 1 μm (F).

less contrast compared with chromosomes during later mitotic stages, digital registration of both the optical and the physical microscopy sections allowed us to discriminate between chromosomes (Figure 3Aiii) and other intracellular structures, including the nucleolus, which was not stained with DAPI. A grayscale threshold was used to confirm the distinction between the chromosomes and nucleolus (prophase chromosomes were 36% darker). Both the chromosomes and the nucleolus were modeled using the SBF SEM dataset (Figure 3B; Figures S3A and S3B; Movie S2).

Segmentation analysis confirmed the presence of 43 discrete units (Figures 3C and 3D), with an average diameter of 0.64 ± 0.19 μm and a combined volume of 109.8 μm^3. Prophase chromosomes exhibited an irregular "lumpy" surface with a total area of 1175 μm^2 (Figures 3Cv and 3Cvi). It is tempting to speculate that the lumps could correspond to topologically associated domains (TADs) or other aspects of interphase chromatin organization that had not yet disassembled in this early prophase cell (Dekker et al., 2013). Because the total DNA content of a dividing cell is 12,344 Mb and the collective chromosome volume is 109.8 μm^3, we calculate that the DNA compaction ratio of prophase chromosomes is 112.4 Mb/μm^3.

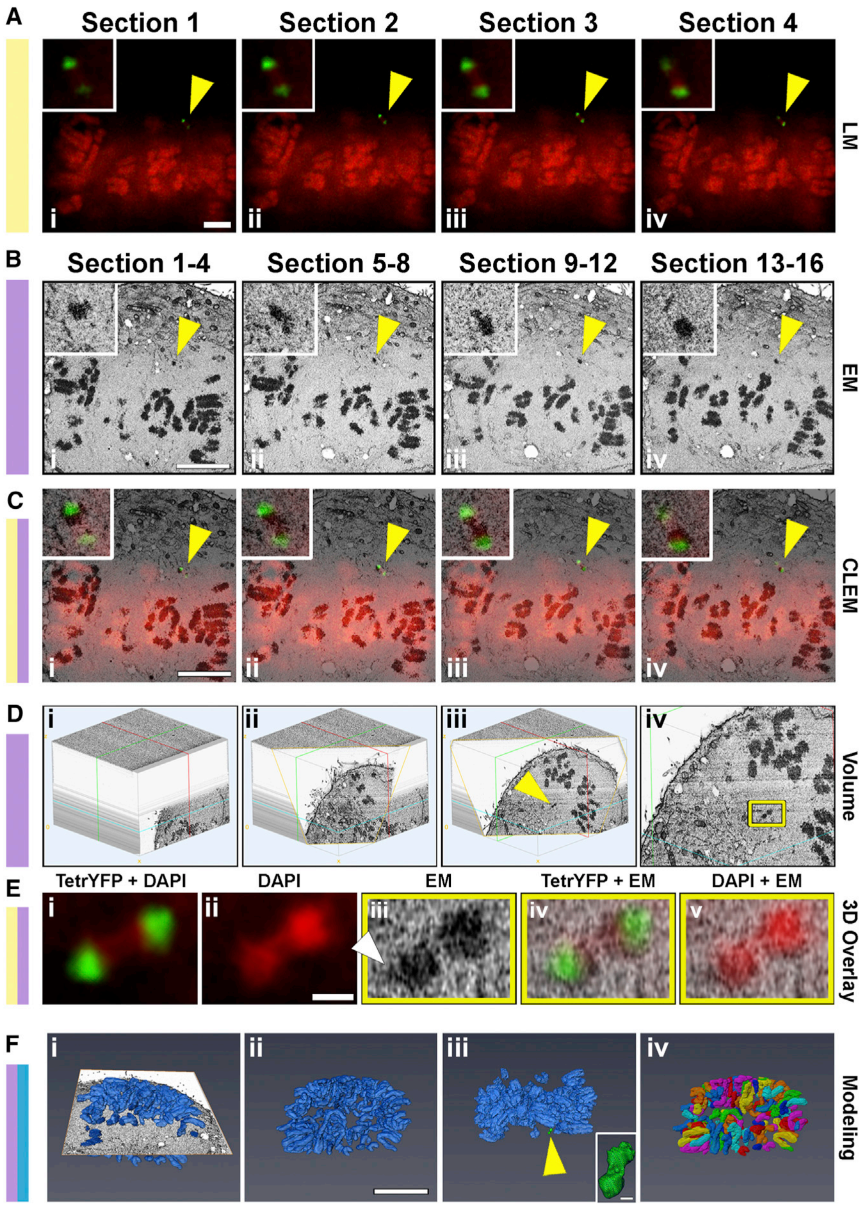

Figure 2. Testing the Utility of 3D-CLEM through Targeted Analysis of the Alphoid^{tetO} HAC

A 1C7 cell containing the HAC was processed for 3D-CLEM.

(A) LM images of a mitotic cell (DAPI) with a visible HAC (tetR-YFP). The HAC (yellow arrow) was present in four optical sections of a complete optical z stack.

(B) SBF SEM images of the HAC. The HAC was present in 16 of the 600 × 60 nm EM sections. To make physical SBF SEM sections and optical LM sections more directly comparable, the 16 SBF SEM sections containing the HAC were collapsed, generating 4 × 240 nm projections.

(C) CLEM registration of both LM and EM sections confirm the location of the HAC (yellow arrows) in all sections shown.

(D) Volume reconstruction of SBF SEM data, using ImageJ *VolumeViewer*. Images show three different cross-sectional positions through the cell (i–iii) including the region of the HAC (iii, yellow arrow, and enlargement in iv).

(E) CLEM registration using LM and EM images. Images show LM projections of DAPI + tetR-YFP (i), DAPI alone (ii), a high-magnification image of the HAC volume (iii), and overlays of LM and EM images (iv and v). Volume analysis of the HAC also revealed the presence of a kinetochore (iii, white arrow).

(F) Modeling and segmentation. Images show an orthoslice with the modeled native chromosomes (i), the modeled natural chromosomes alone (ii), the natural chromosomes plus the HAC (iii, green), and 101 individual, segmented chromosomes (iv).

Scale bars, 3 μm (A); 6 μm (B and C); 500 nm (E); 8 μm (F). See also Figure S1 and Movie S1.

Classic electron microscopy studies revealed that prophase chromosomes form initially next to the nuclear envelope (Robbins and Gonatas, 1964). Indeed, near-complete modeling of the nuclear envelope revealed that 42 of 43 segmented chromosome units make at least one contact with the envelope, with most chromosomes having multiple contacts (Figures S3B and S3C; Movie S2). Because we cannot render the entire nuclear envelope at this resolution, it is possible that all 43 chromosomes make such contacts. These contacts appear to be distributed randomly across the inner nuclear surface. It is possible that the contact points on the chromosomes are LADs (Amendola and van Steensel, 2014); however, we cannot answer this with present technology. The prophase chromosomes are too irregular for us to identify individual chromosomes based on morphological criteria (in contrast with the situation at metaphase; see later). This is a challenge for future technical development.

3D-CLEM of Metaphase Chromosomes

We next imaged a metaphase cell by light microscopy (Figures 4Ai and 4Aii) and subsequently, the Gatan 3View system using the pipeline shown in Figure 1. CLEM registration confirmed the imaging of the chromosomes from the exact same cell (Figures 4Aiii–4Avi, yellow arrows and enlargements). The 3View imaged the complete cell in 300 sections, with chromosomes present between sections 56 and 209 (Figure 4B, yellow arrows). All chromosomes were modeled using semi-automated rendering (Figures 4Cii and 4Di).

Semi-automated segmentation recognized all 46 individual chromosomes (Figures 4Ciii and 4Dii; Movie S3). We unambiguously identified the three largest chromosome pairs (corresponding to chromosomes 1–3) by analyzing their volumes, surface areas, and the position of their primary constrictions (Figures 4D, 4E, and 5A–5D).

Other chromosome groups identified included the submetacentric group B (chromosomes 4 and 5) and several smaller

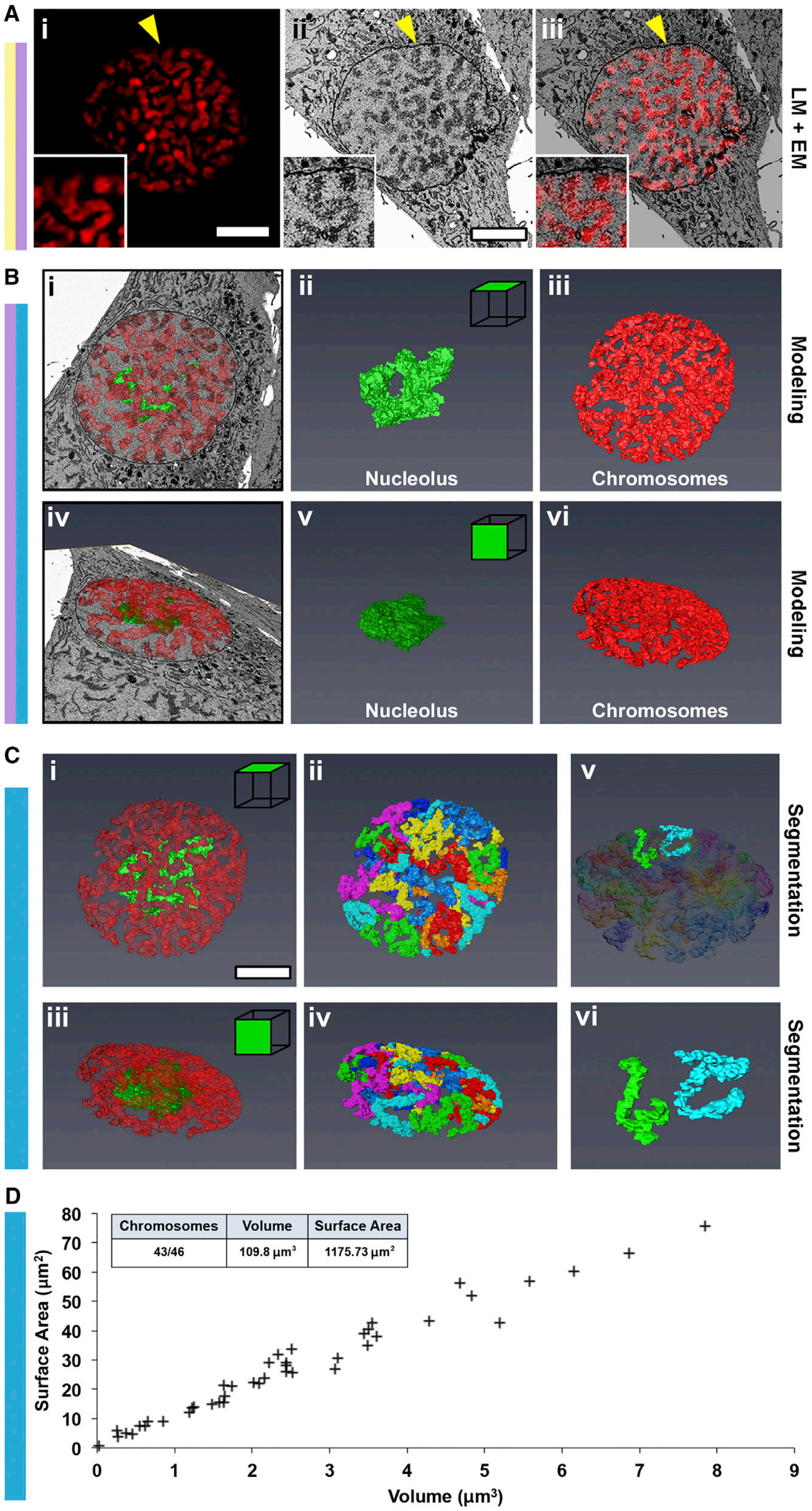

Figure 3. 3D-CLEM Reveals the Architecture of Prophase Chromosomes

(A) A mitotic RPE cell, in prophase, was imaged using DAPI (i), before processing for SBF SEM (ii). The sample was imaged using the 3View system with the cell of interest located using LM images. A single section montage was used for CLEM registration using an optical section and physical EM section (iii). Yellow arrow (and enlargement) shows a clearly registered chromosome.

(B) The nucleolus (green) and chromosomes (red) were modeled using Amira.

(C) Chromosome segmentation. Forty-three of 46 individual chromosomes were successfully separated. Images show the model of the entire chromosome complement (i and iii), separated chromosomes (ii and iv), and an enlargement of two randomly chosen chromosomes (v and vi).

(D) A 2D scatterplot of chromosome volume versus surface area, for all 43 separated chromosomes. Inset shows a summary table of image statistics.

Scale bars, 5 µm (A and C). Magnifications are 2× (A). See also Figure S3 and Movie S2.

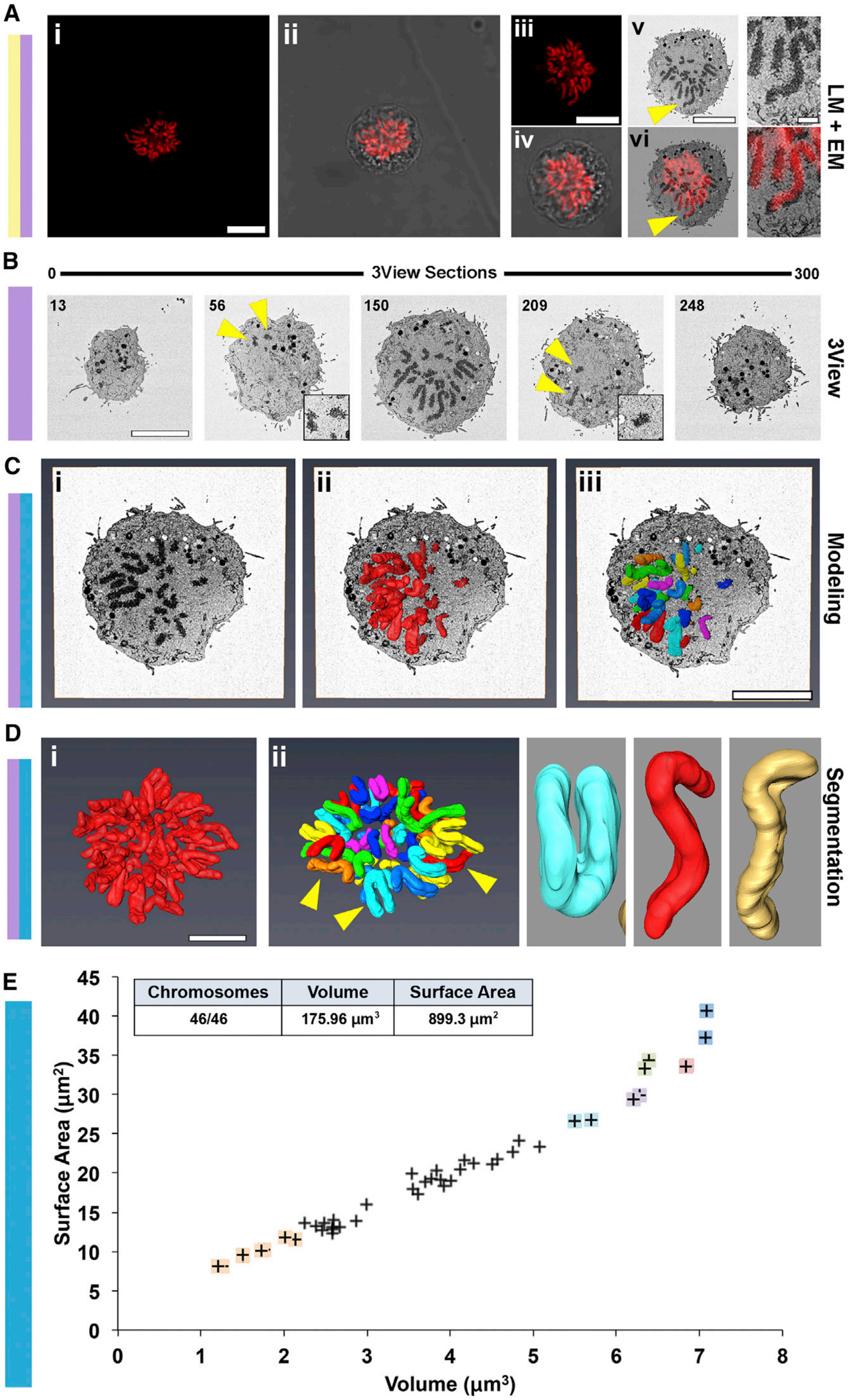

Figure 4. 3D-CLEM of All 46 Human Metaphase Chromosomes

(A) A mitotic RPE cell was identified using DAPI and phase contrast (i and ii) before processing for SBF SEM. The sample was imaged using the 3View system, with the cell of interest located using LM images. A single section montage was used for CLEM registration using an LM optical section (iii and iv), a physical SBF SEM section (v), and an overlay of the two (vi). A clear overlap can be seen between the DAPI- and contrast-stained chromosomes of the EM micrograph. (v and vi) Yellow arrows point to magnified regions of perfect LM and EM registration.

(B) Five of the 300 SBF SEM images acquired. Very early and late sections (13 and 248), the first and last sections to contain chromosomes (56 and 209, yellow arrows), and a middle section where numerous chromosomes are visible (150) are shown.

(C) Chromosome modeling and segmentation with an orthoslice. Images show the orthoslice alone (i), the chromosome complement model traversing the orthoslice (ii), and the segmented chromosomes model traversing the orthoslice (iii).

(D) Chromosome modeling alone. Images show the full chromosome complement (i) and segmented chromosomes (ii). Arrows point to examples of individual chromosomes shown in the zoom panels. These include examples of metacentric (cyan) and submetacentric (red, orange) chromosomes.

(E) A 2D scatterplot of chromosome surface area versus chromosome volume for all 46 chromosomes. Colored marks represent unambiguously characterized chromosomes, including two copies of chromosome 1 (indigo), chromosome 2 (pink), chromosome 3 (green), chromosome 4 (purple), and chromosome 5 (cyan). Two copies of chromosomes 19–22 (peach) were also identified. Inset is a summary table of image statistics.

Scale bars, 10 μm (Ai, Aiii, Av, left panel, B–D), 2 μm (Av, right panel). See also Figure S2 and Movie S3.

chromosomes from groups E, F, and G (Figures 4E and 5D; Table S1).

The chromosome diameter (mean = 1.15 ± 0.12 μm for the paired sister chromatids) was remarkably constant regardless of DNA content (Figures 5B and 5C) and was significantly larger and less variable than that of prophase chromosomes. The total volume occupied by all 46 chromosomes was 175.9 μm^3, with a combined surface area of 899 μm^2, 23% less than that of prophase chromosomes (Figure 4E). This decrease in surface area may arise in part because the surface of mitotic chromosomes is much smoother than that of their prophase counterparts (cf. Figures 3Cvi and 4Dii) and in part because of increased chromatin compaction. Plotting the DNA content (http://www.ensembl.org) of diploid chromosomes that could be unambiguously identified as a function of chromosome volume revealed an almost perfectly linear relationship (Figure 5D), suggesting that chromosome compaction is constant regardless of chromosome shape or size.

A previous electron microscopy study estimated that human mitotic chromosomes were composed of an aggregation of radial loops of 83 kb (Earnshaw and Laemmli, 1983). Given the packing density measured above from full 3D volume measurements, we

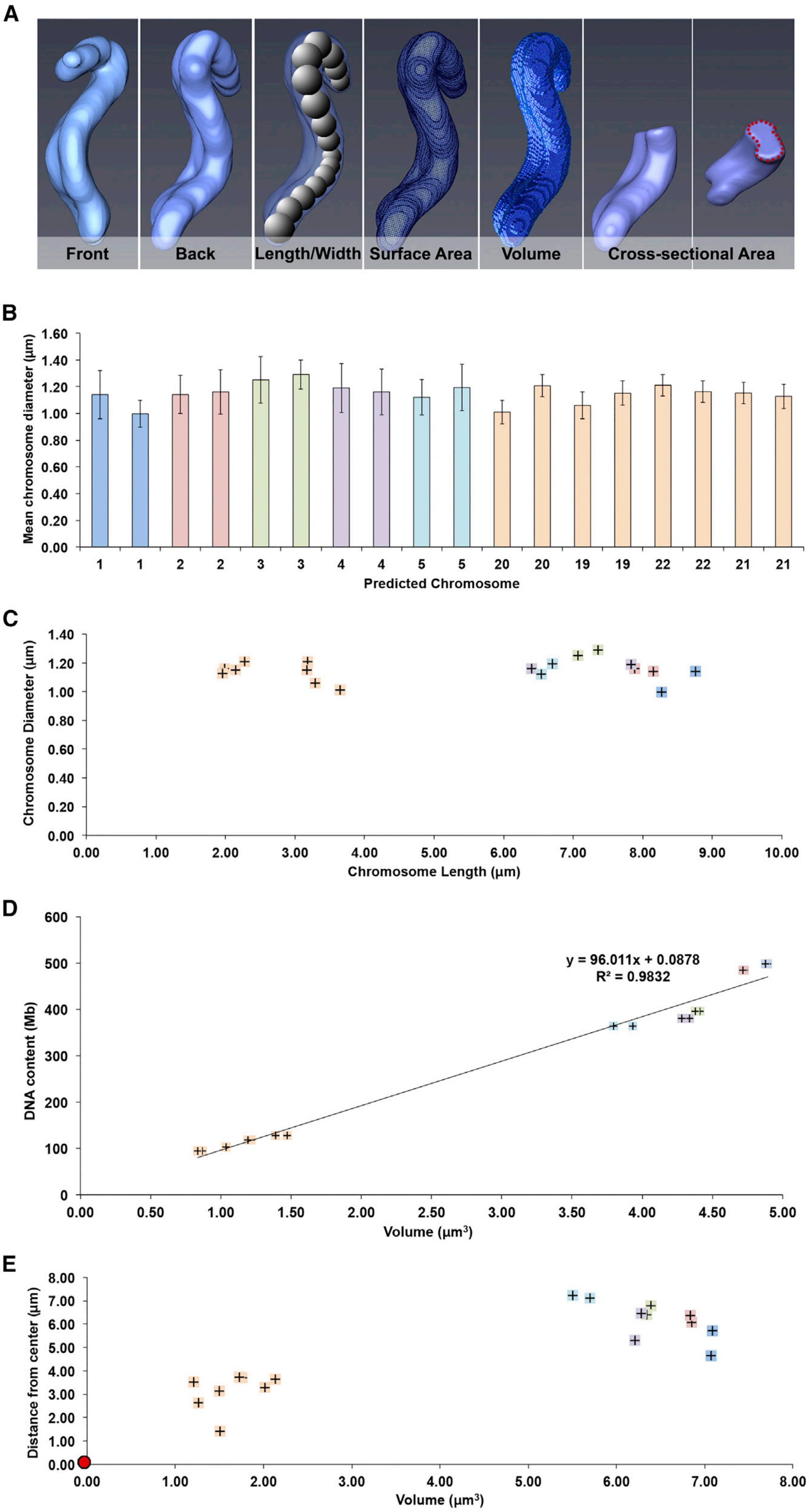

Figure 5. Structural Parameters of the 46 Metaphase Chromosomes Determined by 3D-CLEM

(A) 3D model of human chromosome 4, used to illustrate how chromosome geometry is obtained, for a diploid chromosome.

(B) A bar chart showing mean chromosome width for individual chromosomes 1–5 and 19–22. Error bars represent ±SEM.

(C) 2D scatterplot showing chromosome diameter versus length for chromosomes 1–5 and 19–22.

(D) A 2D scatterplot showing chromosome volume versus DNA content (http://www.ensembl.org) for the unambiguously characterized chromosomes. The calculated y value provides a standard curve formula to estimate chromosome condensation relative to DNA content, and vice versa. The volumes of the metaphase chromosomes have been adjusted to reflect chromosome volume with the periphery subtracted to improve the accuracy of our data (see Table S1 for raw values).

(E) 2D scatterplot showing the radial location within the cell of the mid-point for the chromosomes that could be unambiguously identified (numbers 1–5 and 19–22), relative to the center of the chromosome mass (red dot).

estimate that there are on average 1,040 loops/μm^3 along the condensed mitotic chromosome, or 123,500 loops in total per mitotic cell. This is reasonably similar to the value of 95,000 loops of 63 kb proposed more than 30 years ago (Pienta and Coffey, 1984), based on a literature survey of a number of studies of chromatin domains (Paulson and Laemmli, 1977; Cook and Brazell, 1978; Marsden and Laemmli, 1979).

The availability of these packing data for known chromosomes enabled us to perform an independent estimate of the DNA content of the alphoid^{tetO} HAC. For this, we generated a standard curve by combining chromosome packing data from both metaphase RPE1 and DT40 cells (Figure S1Bi). From the calculated slope of the curve of DNA content versus volume, we estimated the HAC to be ~11.17 Mb, or ~5.58 Mb per sister chromatid (Figure 1Bii, green box). This is very close to the roughly 5 Mb previously estimated by molecular biology methods (Kouprina et al., 2012, 2014). Combining the results from 3D-CLEM and molecular analysis strongly suggests that the packing density of chromatin in the synthetic alphoid^{tetO} HAC and the other native human chromosomes is comparable. Thus, the mechanisms used to form condensed mitotic chromosomes are independent of chromosome shape, size, and possibly even species.

The present analysis of a retinal pigment epithelium (RPE) cell also yielded information about the distribution of metaphase chromosomes within the cell. As suggested by two previous studies (McIntosh and Landis, 1971; Mosgöller et al., 1991), larger chromosomes were positioned toward the cell periphery (average position 6.21 ± 0.8 μm from the cell center), and smaller chromosomes were located more centrally (3.13 ± 0.78 μm) (Figure 5E). The reason for this phenomenon is not known.

Structure of Mitotic Chromosomes Lacking Ki-67

We previously reported that Ki-67, which is recruited to the chromosome periphery in late prophase after nucleolar disassembly (Figure 6A), is required for assembly of much or all of the mitotic chromosome periphery and for keeping chromosomes individualized during mitosis and mitotic exit (Booth et al., 2014). This was confirmed by a recent study, which argued that Ki-67 acts like a biological surfactant on the chromosome surface (Cuylen et al., 2016). To test the role of Ki-67 in mitotic chromosome packing, we performed 3D-CLEM of a metaphase RPE1 cell depleted of Ki-67 (Figures 6B–6E).

We could readily identify and model the Ki-67-depleted chromosomes (Figure 6C), but segmentation analysis identified only 20 individual units, rather than the 46 seen in wild-type cells (Figures 6D and 6E). This confirms the clumping of Ki-67-depleted chromosomes as reported in previous studies (Cuylen et al., 2016; Booth et al., 2014). The total chromosome volume, 170 μm^3 (Figure 6E), was slightly less than that of unperturbed metaphase chromosomes, but light microscope modeling revealed that the DNA occupied a significantly larger volume (Figures S4C and S4D). We calculated (see Supplemental Experimental Procedures) that in these chromosomes the thickness of the periphery layer is likely to be significantly thinner, about 77 nm (roughly half the normal value seen in metaphase cells). This may account for the fact that the surface of the chromosomes looks significantly "rougher" than the surface of unperturbed metaphase chromosomes (Figure 6Diii), and may suggest that there is a small, Ki-67-independent periphery compartment.

This analysis therefore confirmed that Ki-67 is responsible for assembly of a significant portion of the chromosome periphery, and in its absence the mitotic chromatin is slightly less compacted than normal.

Rethinking the Structure of Mitotic Chromosomes

We were extremely surprised to find that the total volume of metaphase chromosomes is 38% greater than that of prophase chromosomes (176 versus 110 μm^3; Figure S4D). This was particularly surprising, given that a recent study of RPE1 cells by light microscopy reported that prophase chromosomes had a significantly larger volume than metaphase chromosomes (450–800 versus 240 μm^3, numbers are extracted from figure 2 in Nagasaka et al., 2016). Indeed, in the present study, the volume of DAPI-stained prophase chromosomes (635 μm^3) was also significantly larger than the metaphase volume (256 μm^3). These values correspond well to the published figures, but they are remarkably different from the chromosome volumes for the same cells determined by electron microscopy (Figure S4).

Because our experiments used CLEM, we could measure the diameter of the identical prophase and metaphase chromosomes in the light and electron microscope (Figure S5). The resulting values were essentially identical.

We can also be confident in the z value in the EM images, because an 18.66 μm metaphase cell was sectioned in 300 sections (~60 nm per section, as specified on the 3View). The volume discrepancy appears to be due to problems with structural modeling of light micrographs in the z direction (where resolution is less). This confounding effect of limiting z of resolution is supported by our attempts to apply the same segmentation parameters used for the EM data to the light microscope data. When segmented by Amira, only 6 individual chromosomes could be recognized in prophase, compared with 43 in the EM (Figures S4A and S4D). This raises the important caveat that volume measurements from light microscopy of complex objects may differ systematically, and significantly from reality for objects where modeling of fine features is required.

Having established that the volume of prophase chromosomes is actually less than that of metaphase, we noted that the volume of the nucleolus (54.4 μm^3) almost exactly explains the difference between the two. All known chromosome periphery proteins, including Ki-67, reside within the nucleolus during interphase and re-localize to the chromosome periphery after nucleolar disassembly in late prophase (Gautier et al., 1992b). If we add the volume of the nucleolus to that of the prophase chromatin, we achieve a final volume of 164 μm^3, which is much closer to the metaphase volume (176 μm^3). Interestingly, if we model the prophase chromosomes as a cylinder ($V = \pi r^2 h$), uniform addition of the nucleolar material to the early prophase chromatin would generate a perichromosomal layer 70 nm thick (Supplemental Experimental Procedures). This is in remarkable agreement with the recent observation that the Ki-67 layer on mitotic chromosomes is 87 nm thick (Cuylen et al., 2016).

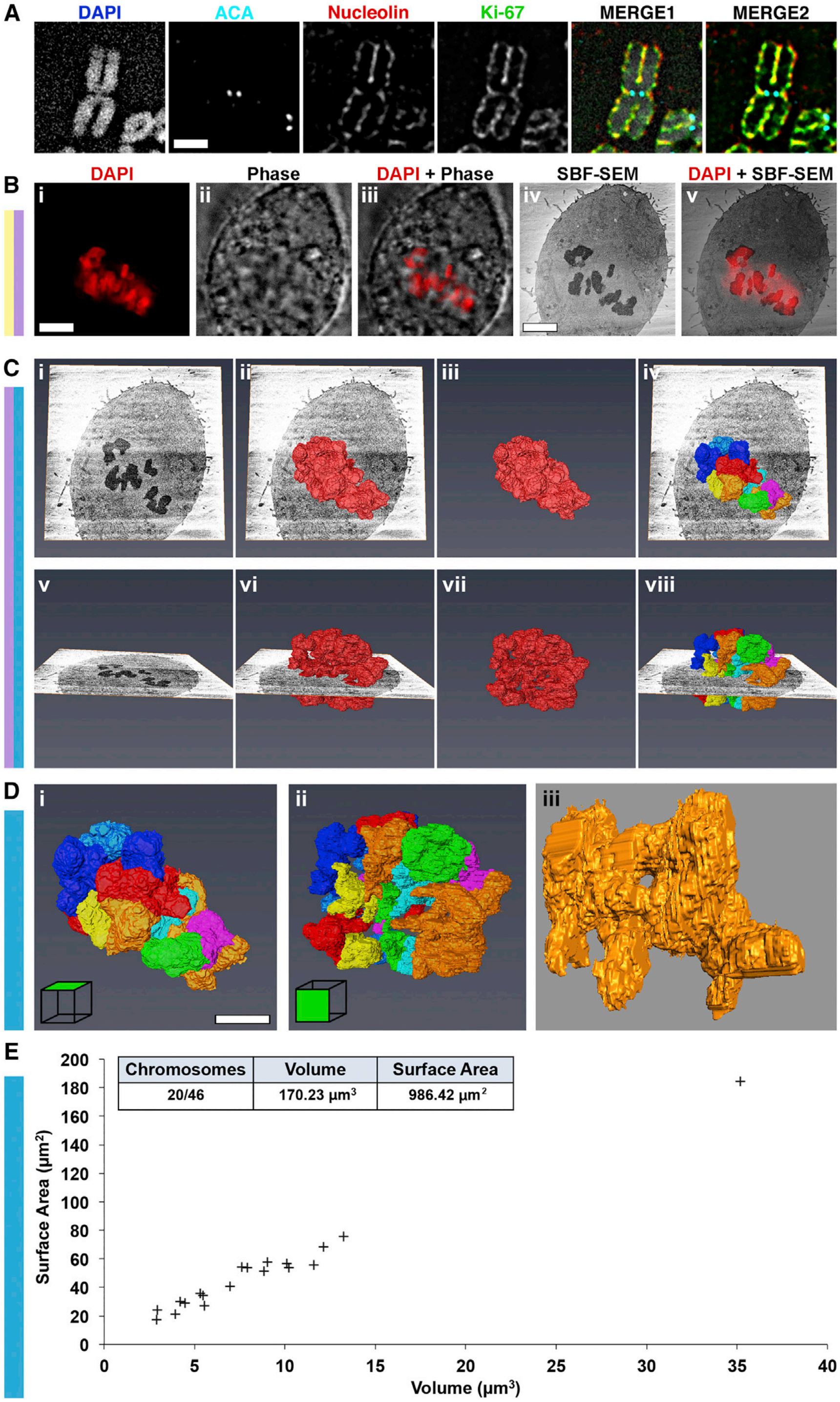

Figure 6. 3D-CLEM of Metaphase Chromosomes Depleted of Ki-67

(A) Light microscopy and visualization of the chromosome periphery on RPE metaphase chromosomes using antibodies against anti-centromere antibody (ACA), nucleolin, and Ki-67.

(B) A mitotic RPE cell, depleted of Ki-67 using siRNA, was identified using DAPI and phase contrast (i–iii) before processing for SBF SEM (iv). The sample was imaged using the 3View system and the cell of interest located using LM images. A single section montage was used for CLEM registration using an LM optical section and a physical SBF SEM section. An overlay of the two is shown (v).

(C) Chromosome modeling and segmentation with an orthoslice. Images show the orthoslice alone (i and v), the chromosome complement model traversing the orthoslice (ii and vi), the chromosome complement model alone (iii and vii), and the segmented chromosomes model traversing the orthoslice (iv and viii).

(D) Segmented chromosomes. Images show model of segmented chromosomes (i and ii) and an enlargement of one randomly chosen chromosome cluster (iii).

(E) A 2D scatterplot of chromosome volume versus surface area, for all successfully separated chromosomes. Inset is a summary table of image statistics.

Scale bars, 1 μm (A); 5 μm (B and D).

Previous measurements of the thickness of the periphery layer on metaphase chromosomes have ranged from 143 nm (our measurements from the micrographs of Gautier et al., 1992a) to 160 nm (Booth et al., 2014). If we model the chromosomes as a cylinder and include a periphery compartment of 150 nm in the volume, we find that the chromatin volume of metaphase chromosomes is actually 15% less than that of the prophase chromosomes determined here (calculations in Supplemental Experimental Procedures). This suggests that much of mitotic chromatin compaction is actually completed by early prophase, and that subsequent changes in morphology involve primarily remodeling (primarily shortening and thickening) of the structure, resulting in a further 15% compaction.

An extremely surprising conclusion from this analysis is that a very substantial percentage of mitotic chromosomes is not composed of chromatin. On a volume basis, we calculate that from 30% to 47% of the chromosome volume is actually in the periphery compartment. This sounds counterintuitive, but volume scales with the cube of the radius, so a relatively thin surface layer contains a substantial proportion of the total volume.

Detailed analysis of proteomic data backs up the surprising conclusion that mitotic chromosomes are only 53%–70% chromatin (Ohta et al., 2010; Samejima et al., 2015). We conducted a clustering and correlation analysis of our total proteomic data (Figure 7) and could identify two major clusters corresponding to nucleolar and known periphery proteins (red) and ribosomal and RNA-associated proteins (purple). Ki-67 (red) occupied a separate position in the analysis. Using the intensity-based

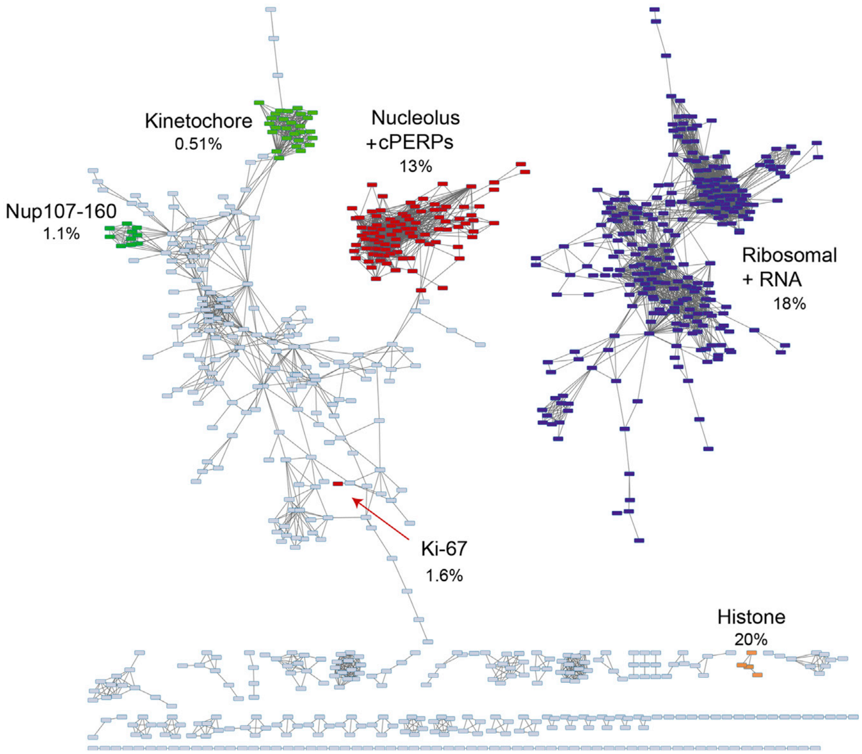

Figure 7. Estimating the Protein Mass of the Chromosome Periphery with Mass Spectrometry

Correlation analysis between all pairs of proteins associated with chromosomes across the experiments that were reported in Samejima et al. (2015). The network analysis revealed sub-complexes as mutually connected proteins. cPERPs (ABDEF) were found among the network, which contains many nucleolar proteins. This was distantly connected with the rest of the chromosomal complexes. Ribosomal proteins and other proteins involved in RNA metabolism such as EIF3, mediator, splicing factors, THO-TREX complex, and RNA polymerase II constituted a distinct network. Golgi components are also in this network and may well also be chromosome peripheral components.

absolute quantification (iBAQ) routine in MaxQuant, we determined that 33% of the entire chromosomal protein mass falls in these clusters. Ki-67 itself constitutes a remarkable 1.6% of the total chromosomal protein mass. For comparison, core histones make up 20% of the total protein mass. Bearing in mind that the chromosome periphery is likely to also be rich in pre-rRNA and small nucleolar RNAs (snoRNAs), this analysis is entirely consistent with our morphology-based calculations that the periphery constitutes 30%–47% of the chromosome volume.

Conclusions

Combining CLEM with 3D rendering in the 3View or comparable FIB-SEM microscopes (Kizilyaprak et al., 2014) now permits the three-dimensional analysis of chromosomes at different phases of mitosis, as well as the structures of particular chromosomes that can be recognized, either as a consequence of their volume, characteristic morphology, or after the binding of fluorescent marker proteins. This first application of 3D-CLEM analysis yielded the remarkable, and surprising, conclusion that a very large percentage of the total volume of mitotic chromosomes is not composed of chromatin, but is instead in the periphery compartment. We and others have shown that this compartment is at least partly assembled as a result of Ki-67 binding to the chromosome surface, where it is required to keep sister chromatids separate (Booth et al., 2014; Cuylen et al., 2016). The behavior of this compartment, for example, whether it is liquid-like (Berry et al., 2015; Brangwynne et al., 2011), and how it influences the structural changes in the chromatin during the transition from interphase to mitosis (Naumova et al., 2013) will now become a much more active area of study.

EXPERIMENTAL PROCEDURES

Cell Culture and Transfection

RPE1-hTERT and 1C7 cells (Cardinale et al., 2009) were maintained in DMEM (Invitrogen) supplemented with 5% fetal bovine serum (FBS) (Invitrogen) and 100 U/mL penicillin G and 100 μg/mL streptomycin sulfate (Invitrogen). Blasticidin S (Invitrogen) was added to a growing culture of 1C7 cells at a final concentration of 4 μg/mL to maintain the alphoid^{tetO} HAC. 1C7 cells were transfected with a plasmid for expressing the tetR-EYFP fusion protein using the XtremeGene9 reagent (Roche) following manufacturer's instructions. RPE cells were transfected with siRNA targeting Ki-67 as previously described (Booth et al., 2014).

Chromosome Spreads and Immunostaining Analysis

Chromosome spreads from RPE1-hTERT cells were prepared after treatment of cells with 0.2 μg/mL colcemid for 3 hr before harvesting. After trypsinization, cells were treated with hypotonic solution (0.075M KCl) for 20 min at 37°C and fixed with Carnoy solution (methanol/acetic acid 3:1).

Immunofluorescence (IF) staining of unfixed metaphase chromosomes was performed as previously described (Bergmann et al., 2011; Molina et al., 2012). See Supplemental Experimental Procedures for more details.

Preparation of Cells for SBF SEM

Cells were seeded onto gridded dishes (MatTek) and fixed with 3% glutaraldehyde and 1% paraformaldehyde in 0.1 M sodium cacodylate buffer for 1 hr at room temperature (RT). Cells were then washed with PBS 3 × 5 min; one PBS wash contained Hoechst 1:1,000. Cells were imaged by light microscopy using a DeltaVision wide-field microscope. Samples were prepared for SBF SEM, based on the Deerinck and Ellisman protocol (West et al., 2010). Extra contrasting steps were introduced, compared with those used for standard TEM to reduce charging and improve the signal-to-noise ratio. In detail, after fixation and imaging with light microscopy, the cells were postfixed and stained with reduced osmium (2% osmium tetroxide in dH_2O + 1.5% potassium ferrocyanide in 0.1 M sodium cacodylate buffer) for 1 hr at RT. This was followed by 0.1% tannic acid in ddH_2O for 20 min at RT. Tannic acid acts as a mordant, facilitating binding of heavy metals to biological structures (alternatively 1% thiocarbohydrazide can be used, which enhances membrane staining compared with cytoplasmic staining by tannic acid). A second osmication step (2% in ddH_2O for 40 min at RT) preceded an overnight incubation in aqueous 1% uranyl acetate at 4°C. The next day cells were stained with

Walton's lead aspartate (0.02 M in lead nitrate + 0.03 M in aspartic acid in ddH_2O, adjusted to pH 5.5) for 30 min at 60°C. To prevent precipitation artifacts, we washed the cells for a minimum of 5 × 3 min with ddH_2O between each of the staining steps described. Next, samples were dehydrated in a graded ethanol series of 30%, 50%, 70%, and 90% in ddH_2O for 5 min each, followed by 2 × 5 min 100% ethanol. Samples were then infiltrated with TAAB Hard Premix resin at ratios of 1:1, 2:1, and 3:1 with resin:100% ethanol, 30 min per incubation. Finally, samples were incubated in 100% resin for 2 × 30 min, before embedding the whole dish in 2 mm of 100% fresh resin. Samples were cured for 48 hr at 60°C.

Preparation of Blocks for 3View SBF SEM

Resin is separated from the gridded dish by trimming away the excess plastic and carefully sliding a razor between the dish and the resin (Booth et al., 2013). Excess resin is removed using a junior hacksaw and scalpel before the block is mounted onto a cryo pin, cell side up, using superglue or a non-conductive compound. Targeted trimming is performed using an ultra-microtome and etched coordinates (Booth et al., 2013).

SBF SEM Imaging and Acquisition

Samples were painted with Electrodag silver paint (avoiding the block face) and then coated with 10 nm AuPd using a Q150T sputter coater (Quorum Technologies). The sample was inserted into the Gatan 3View sample holder and adjusted so the block face would be central in the microtome and parallel with the knife-edge. After loading into the Gatan 3View microtome, the sample height was raised manually until the block face was close to the height of the knife. The final approach of the block face to the knife was achieved by attaching the dissection microscope to the 3View door and using the *automatic approach* on Digital Micrograph, at 200 nm thick slices.

Imaging a single cell creates challenges because the resin surrounding the cell is non-conductive. Without a conductive escape path for the electrons, charging builds up, the resin softens, and this causes distortion of the block face with each subsequent slice. To mitigate charge buildup and maximize image quality, imaging conditions must be finely balanced. Cells were imaged in low vacuum mode with a chamber pressure of 50 Pa. Low accelerating voltage (2.5 kV) was also used to reduce charging; however, this results in lower detector efficiency, which was compensated for with a slower dwell time per pixel (60 μs). Ultimately, a suitable magnification (3,875×) was determined by the predicted size of the cell at its widest point (~20 μm). To obtain a typical resolution of 24 nm in x and y, a frame width of 1024 × 1024 was used. Section thickness was 60 nm over 200–600 sections, depending on the cell type. 1 × 1C7, 2 × DT40, and 5 × RPE1-hTERT cells were imaged.

3D Reconstruction, Modeling, and Segmentation

3View EM stacks were annotated using Amira (FEI). CLEM registration was performed using primary (EM) and secondary (LM, deconvolved) overlays with the *multiplanar* tool.

Chromosomes present in every orthoslice were annotated using *masking* and *thresholding* alone (fully automated) or in combination with *magic wand* and *blow* tools (semiautomated).

The modeled complement of chromosomes was segmented into discernable isolated objects using *interactive thresholding* and *separate objects* modules. Objects were separated using 3D interpretation and a neighborhood criteria of 26 connected elements, by at least one corner, edge, or face. The marker contrast range (*H-extrema*) was set between 5 and 7, depending on the sample. *Label analysis* modules were used to measure the geometry of all isolated structures. Surface renders were generated using unconstrained smoothing at levels 5–7.

To ascertain the positions of chromosomes within mitotic cells, we defined the position of a chromosome as being the distance between the midpoint of the chromosome (along its length) and the centroid of the cell, as stipulated by the Amira software. We did not use the position of the centromere because this could not be recognized for every chromosome in the reconstructions.

SUPPLEMENTAL INFORMATION

Supplemental Information includes Supplemental Experimental Procedures, five figures, one table, and three movies and can be found with this article online at http://dx.doi.org/10.1016/j.molcel.2016.10.009.

AUTHOR CONTRIBUTIONS

Conceptualization, D.G.B. and W.C.E.; Methodology, D.G.B.; Investigation, D.G.B., A.J.B., O.M., and I.S.; Formal Analysis, D.G.B. and W.C.E.; Writing – Original Draft, D.G.B.; Writing – Review & Editing, D.G.B. and W.C.E.; Funding Acquisition, I.A.P. and W.C.E.; Resources, D.G.B., H.M., N.K., V.L., and I.A.P.; Supervision, D.G.B., I.A.P., and W.C.E.

ACKNOWLEDGMENTS

We thank Leonid Mirny for helpful discussions and suggestions. This work was supported by The Wellcome Trust, of which W.C.E. is a Principal Research Fellow (grant number 073915). I.A.P. gratefully acknowledges support from NWCR. The Wellcome Trust Centre for Cell Biology is supported by Wellcome Trust core grants 077707 and 092076.

Received: April 4, 2016
Revised: August 24, 2016
Accepted: October 5, 2016
Published: November 10, 2016

REFERENCES

Adolphs, K.W., Cheng, S.M., Paulson, J.R., and Laemmli, U.K. (1977). Isolation of a protein scaffold from mitotic HeLa cell chromosomes. Proc. Natl. Acad. Sci. USA *74*, 4937–4941.

Amendola, M., and van Steensel, B. (2014). Mechanisms and dynamics of nuclear lamina-genome interactions. Curr. Opin. Cell Biol. *28*, 61–68.

Bajer, A. (1959). Change of length and volume of mitotic chromosomes in living cells. Hereditas *45*, 579–596.

Belmont, A.S. (2002). Mitotic chromosome scaffold structure: new approaches to an old controversy. Proc. Natl. Acad. Sci. USA *99*, 15855–15857.

Belmont, A.S. (2006). Mitotic chromosome structure and condensation. Curr. Opin. Cell Biol. *18*, 632–638.

Belmont, A.S., Sedat, J.W., and Agard, D.A. (1987). A three-dimensional approach to mitotic chromosome structure: evidence for a complex hierarchical organization. J. Cell Biol. *105*, 77–92.

Bergmann, J.H., Rodríguez, M.G., Martins, N.M., Kimura, H., Kelly, D.A., Masumoto, H., Larionov, V., Jansen, L.E., and Earnshaw, W.C. (2011). Epigenetic engineering shows H3K4me2 is required for HJURP targeting and CENP-A assembly on a synthetic human kinetochore. EMBO J. *30*, 328–340.

Berry, J., Weber, S.C., Vaidya, N., Haataja, M., and Brangwynne, C.P. (2015). RNA transcription modulates phase transition-driven nuclear body assembly. Proc. Natl. Acad. Sci. USA *112*, E5237–E5245.

Booth, D.G., Hood, F.E., Prior, I.A., and Royle, S.J. (2011). A TACC3/ch-TOG/clathrin complex stabilises kinetochore fibres by inter-microtubule bridging. EMBO J. *30*, 906–919.

Booth, D.G., Cheeseman, L.P., Prior, I.A., and Royle, S.J. (2013). Studying kinetochore-fiber ultrastructure using correlative light-electron microscopy. Methods Cell Biol. *115*, 327–342.

Booth, D.G., Takagi, M., Sanchez-Pulido, L., Petfalski, E., Vargiu, G., Samejima, K., Imamoto, N., Ponting, C.P., Tollervey, D., Earnshaw, W.C., and Vagnarelli, P. (2014). Ki-67 is a PP1-interacting protein that organises the mitotic chromosome periphery. eLife *3*, e01641.

Boy de la Tour, E., and Laemmli, U.K. (1988). The metaphase scaffold is helically folded: sister chromatids have predominantly opposite helical handedness. Cell *55*, 937–944.

Brangwynne, C.P., Mitchison, T.J., and Hyman, A.A. (2011). Active liquid-like behavior of nucleoli determines their size and shape in Xenopus laevis oocytes. Proc. Natl. Acad. Sci. USA *108*, 4334–4339.

Cardinale, S., Bergmann, J.H., Kelly, D., Nakano, M., Valdivia, M.M., Kimura, H., Masumoto, H., Larionov, V., and Earnshaw, W.C. (2009). Hierarchical inactivation of a synthetic human kinetochore by a chromatin modifier. Mol. Biol. Cell *20*, 4194–4204.

Chaly, N., Bladon, T., Setterfield, G., Little, J.E., Kaplan, J.G., and Brown, D.L. (1984). Changes in distribution of nuclear matrix antigens during the mitotic cell cycle. J. Cell Biol. *99*, 661–671.

Choo, K.H., Vissel, B., Nagy, A., Earle, E., and Kalitsis, P. (1991). A survey of the genomic distribution of alpha satellite DNA on all the human chromosomes, and derivation of a new consensus sequence. Nucleic Acids Res. *19*, 1179–1182.

Cook, P.R., and Brazell, I.A. (1978). Spectrofluorometric measurement of the binding of ethidium to superhelical DNA from cell nuclei. Eur. J. Biochem. *84*, 465–477.

Cuylen, S., Blaukopf, C., Politi, A.Z., Müller-Reichert, T., Neumann, B., Poser, I., Ellenberg, J., Hyman, A.A., and Gerlich, D.W. (2016). Ki-67 acts as a biological surfactant to disperse mitotic chromosomes. Nature *535*, 308–312.

Dekker, J., Marti-Renom, M.A., and Mirny, L.A. (2013). Exploring the three-dimensional organization of genomes: interpreting chromatin interaction data. Nat. Rev. Genet. *14*, 390–403.

DuPraw, E.J. (1966). Evidence for a 'folded-fibre' organization in human chromosomes. Nature *209*, 577–581.

Earnshaw, W.C., and Laemmli, U.K. (1983). Architecture of metaphase chromosomes and chromosome scaffolds. J. Cell Biol. *96*, 84–93.

Eltsov, M., Maclellan, K.M., Maeshima, K., Frangakis, A.S., and Dubochet, J. (2008). Analysis of cryo-electron microscopy images does not support the existence of 30-nm chromatin fibers in mitotic chromosomes in situ. Proc. Natl. Acad. Sci. USA *105*, 19732–19737.

Finch, J.T., and Klug, A. (1976). Solenoidal model for superstructure in chromatin. Proc. Natl. Acad. Sci. USA *73*, 1897–1901.

Flemming, W. (1882). Zellsubstanz, kern und zelltheilung (F.C.W. Vogel).

Gautier, T., Masson, C., Quintana, C., Arnoult, J., and Hernandez-Verdun, D. (1992a). The ultrastructure of the chromosome periphery in human cell lines. An in situ study using cryomethods in electron microscopy. Chromosoma *101*, 502–510.

Gautier, T., Robert-Nicoud, M., Guilly, M.N., and Hernandez-Verdun, D. (1992b). Relocation of nucleolar proteins around chromosomes at mitosis. A study by confocal laser scanning microscopy. J. Cell Sci. *102*, 729–737.

Hernandez-Verdun, D., and Gautier, T. (1994). The chromosome periphery during mitosis. BioEssays *16*, 179–185.

Kireeva, N., Lakonishok, M., Kireev, I., Hirano, T., and Belmont, A.S. (2004). Visualization of early chromosome condensation: a hierarchical folding, axial glue model of chromosome structure. J. Cell Biol. *166*, 775–785.

Kizilyaprak, C., Daraspe, J., and Humbel, B.M. (2014). Focused ion beam scanning electron microscopy in biology. J. Microsc. *254*, 109–114.

Kouprina, N., Samoshkin, A., Erliandri, I., Nakano, M., Lee, H.S., Fu, H., Iida, Y., Aladjem, M., Oshimura, M., Masumoto, H., et al. (2012). Organization of synthetic alphoid DNA array in human artificial chromosome (HAC) with a conditional centromere. ACS Synth. Biol. *1*, 590–601.

Kouprina, N., Tomilin, A.N., Masumoto, H., Earnshaw, W.C., and Larionov, V. (2014). Human artificial chromosome-based gene delivery vectors for biomedicine and biotechnology. Expert Opin. Drug Deliv. *11*, 517–535.

Kschonsak, M., and Haering, C.H. (2015). Shaping mitotic chromosomes: from classical concepts to molecular mechanisms. BioEssays *37*, 755–766.

Kuwada, Y. (1939). Chromosome structure. A critical review. Cytologia (Tokyo) *10*, 213–256.

Liang, Z., Zickler, D., Prentiss, M., Chang, F.S., Witz, G., Maeshima, K., and Kleckner, N. (2015). Chromosomes progress to metaphase in multiple discrete steps via global compaction/expansion cycles. Cell *161*, 1124–1137.

Luger, K., Mäder, A.W., Richmond, R.K., Sargent, D.F., and Richmond, T.J. (1997). Crystal structure of the nucleosome core particle at 2.8 A resolution. Nature *389*, 251–260.

Marko, J.F. (2008). Micromechanical studies of mitotic chromosomes. Chromosome Res. *16*, 469–497.

Marsden, M.P., and Laemmli, U.K. (1979). Metaphase chromosome structure: evidence for a radial loop model. Cell *17*, 849–858.

McIntosh, J.R., and Landis, S.C. (1971). The distribution of spindle microtubules during mitosis in cultured human cells. J. Cell Biol. *49*, 468–497.

McKeon, F.D., Tuffanelli, D.L., Kobayashi, S., and Kirschner, M.W. (1984). The redistribution of a conserved nuclear envelope protein during the cell cycle suggests a pathway for chromosome condensation. Cell *36*, 83–92.

Molina, O., Blanco, J., Anton, E., Vidal, F., and Volpi, E.V. (2012). High-resolution fish on DNA fibers for low-copy repeats genome architecture studies. Genomics *100*, 380–386.

Mosgöller, W., Leitch, A.R., Brown, J.K., and Heslop-Harrison, J.S. (1991). Chromosome arrangements in human fibroblasts at mitosis. Hum. Genet. *88*, 27–33.

Nagasaka, K., Hossain, M.J., Roberti, M.J., Ellenberg, J., and Hirota, T. (2016). Sister chromatid resolution is an intrinsic part of chromosome organization in prophase. Nat. Cell Biol. *18*, 692–699.

Nakano, M., Cardinale, S., Noskov, V.N., Gassmann, R., Vagnarelli, P., Kandels-Lewis, S., Larionov, V., Earnshaw, W.C., and Masumoto, H. (2008). Inactivation of a human kinetochore by specific targeting of chromatin modifiers. Dev. Cell *14*, 507–522.

Naumova, N., Imakaev, M., Fudenberg, G., Zhan, Y., Lajoie, B.R., Mirny, L.A., and Dekker, J. (2013). Organization of the mitotic chromosome. Science *342*, 948–953.

Nishino, Y., Eltsov, M., Joti, Y., Ito, K., Takata, H., Takahashi, Y., Hihara, S., Frangakis, A.S., Imamoto, N., Ishikawa, T., and Maeshima, K. (2012). Human mitotic chromosomes consist predominantly of irregularly folded nucleosome fibres without a 30-nm chromatin structure. EMBO J. *31*, 1644–1653.

Ohnuki, Y. (1968). Structure of chromosomes. I. Morphological studies of the spiral structure of human somatic chromosomes. Chromosoma *25*, 402–428.

Ohta, S., Bukowski-Wills, J.C., Sanchez-Pulido, L., Alves, Fde.L., Wood, L., Chen, Z.A., Platani, M., Fischer, L., Hudson, D.F., Ponting, C.P., et al. (2010). The protein composition of mitotic chromosomes determined using multiclassifier combinatorial proteomics. Cell *142*, 810–821.

Paulson, J.R., and Laemmli, U.K. (1977). The structure of histone-depleted metaphase chromosomes. Cell *12*, 817–828.

Pienta, K.J., and Coffey, D.S. (1984). A structural analysis of the role of the nuclear matrix and DNA loops in the organization of the nucleus and chromosome. J. Cell Sci. Suppl. *1*, 123–135.

Rattner, J.B., and Lin, C.C. (1985). Radial loops and helical coils coexist in metaphase chromosomes. Cell *42*, 291–296.

Robbins, E., and Gonatas, N.K. (1964). The ultrastructure of a mammalian cell during the mitotic cycle. J. Cell Biol. *21*, 429–463.

Samejima, I., Spanos, C., Alves, Fde.L., Hori, T., Perpelescu, M., Zou, J., Rappsilber, J., Fukagawa, T., and Earnshaw, W.C. (2015). Whole-proteome genetic analysis of dependencies in assembly of a vertebrate kinetochore. J. Cell Biol. *211*, 1141–1156.

Scholzen, T., Endl, E., Wohlenberg, C., van der Sar, S., Cowell, I.G., Gerdes, J., and Singh, P.B. (2002). The Ki-67 protein interacts with members of the heterochromatin protein 1 (HP1) family: a potential role in the regulation of higher-order chromatin structure. J. Pathol. *196*, 135–144.

Sedat, J., and Manuelidis, L. (1978). A direct approach to the structure of eukaryotic chromosomes. Cold Spring Harb. Symp. Quant. Biol. *42*, 331–350.

Strukov, Y.G., Wang, Y., and Belmont, A.S. (2003). Engineered chromosome regions with altered sequence composition demonstrate hierarchical

large-scale folding within metaphase chromosomes. J. Cell Biol. *162*, 23–35.

Swedlow, J.R., and Hirano, T. (2003). The making of the mitotic chromosome: modern insights into classical questions. Mol. Cell *11*, 557–569.

Van Hooser, A.A., Yuh, P., and Heald, R. (2005). The perichromosomal layer. Chromosoma *114*, 377–388.

West, J.B., Fu, Z., Deerinck, T.J., Mackey, M.R., Obayashi, J.T., and Ellisman, M.H. (2010). Structure-function studies of blood and air capillaries in chicken lung using 3D electron microscopy. Respir. Physiol. Neurobiol. *170*, 202–209.

Whitfield, M.L., George, L.K., Grant, G.D., and Perou, C.M. (2006). Common markers of proliferation. Nat. Rev. Cancer *6*, 99–106.

Molecular Cell, Volume *64*

Supplemental Information

3D-CLEM Reveals that a Major Portion of Mitotic Chromosomes Is Not Chromatin

Daniel G. Booth, Alison J. Beckett, Oscar Molina, Itaru Samejima, Hiroshi Masumoto, Natalay Kouprina, Vladimir Larionov, Ian A. Prior, and William C. Earnshaw

Supplementary Figure 1 (related to Figure 2)

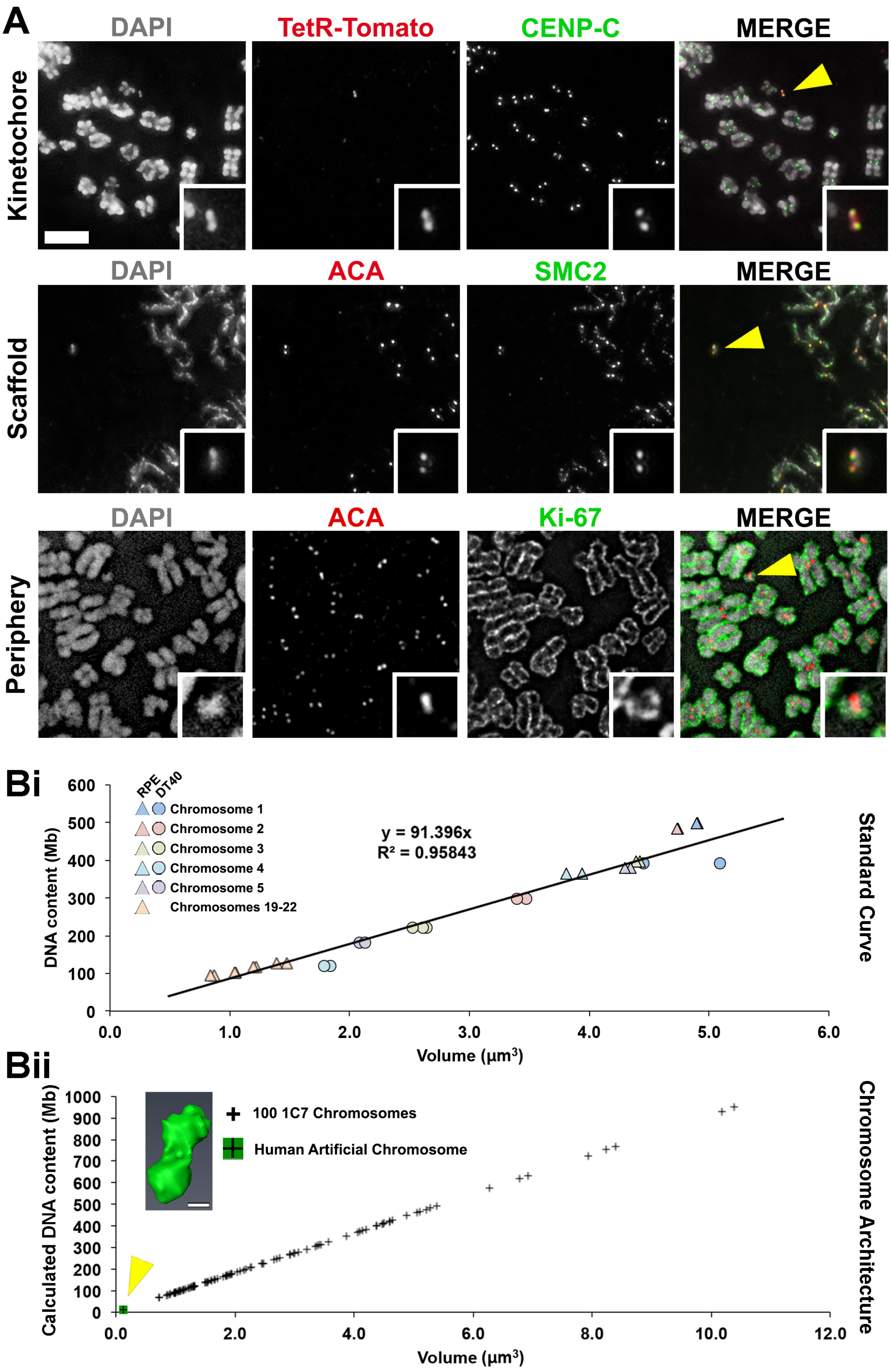

Supplementary Fig. 1: A human artificial chromosome (HAC) contains components from all major chromosome compartments. A) 1C7 cells were used for metaphase spreads. Chromosomes were probed with antibodies targeting major chromosome compartments: The kinetochore (CENP-C), the chromosome scaffold (SMC2) and the chromosome periphery (Ki-67). DAPI, human anti-centromere autoantibodies (ACA) or TetR-tomato were used to identify the HAC. Bi) A scatter plot of volume versus DNA content for chromosomes 1-5 from both RPE (triangles) and DT40 (circles) cells analyzed in Figs. 1 and 4. Line of best fit allows Y value to be determined. Bii) A 2D plot of volume versus DNA content for 1C7 HAC data. Using the standard curve from Suppl Fig. 1Bi, DNA content of all 1C7 chromosomes was estimated, using chromosome volume as the known variable. The DNA content of the HAC (green box and image, inset) was also predicted. Bar = A - 4 µm, H – 250 nm.

Supplementary Figure 2 (related to Figure 4)

A

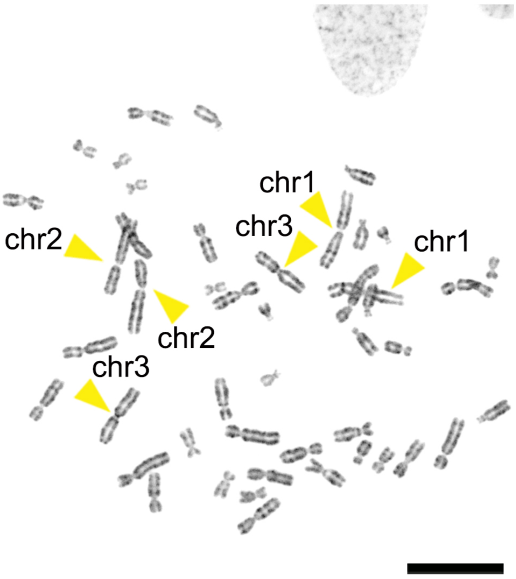

B

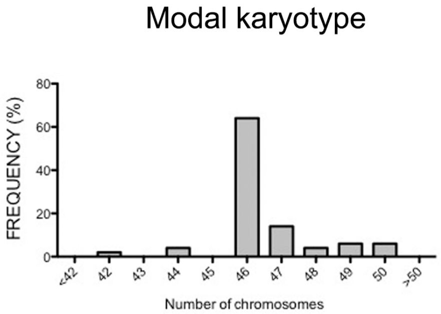

Supplementary Fig. 2: RPE1-hTERT cells maintain a stable modal karyotype of 46 chromosomes. A) Representative RPE1-hTERT metaphase chromosome spread. Chromosomes are stained with DAPI. Yellow arrowheads point to chromosomes 1, 2 and 3 identified by DAPI banding. **B)** Modal karyotype of the RPE1-hTERT cell line. The histogram shows the frequency in which different chromosome numbers were observed in 50 individual metaphases.

Supplementary Figure 3 (related to Figure 3)

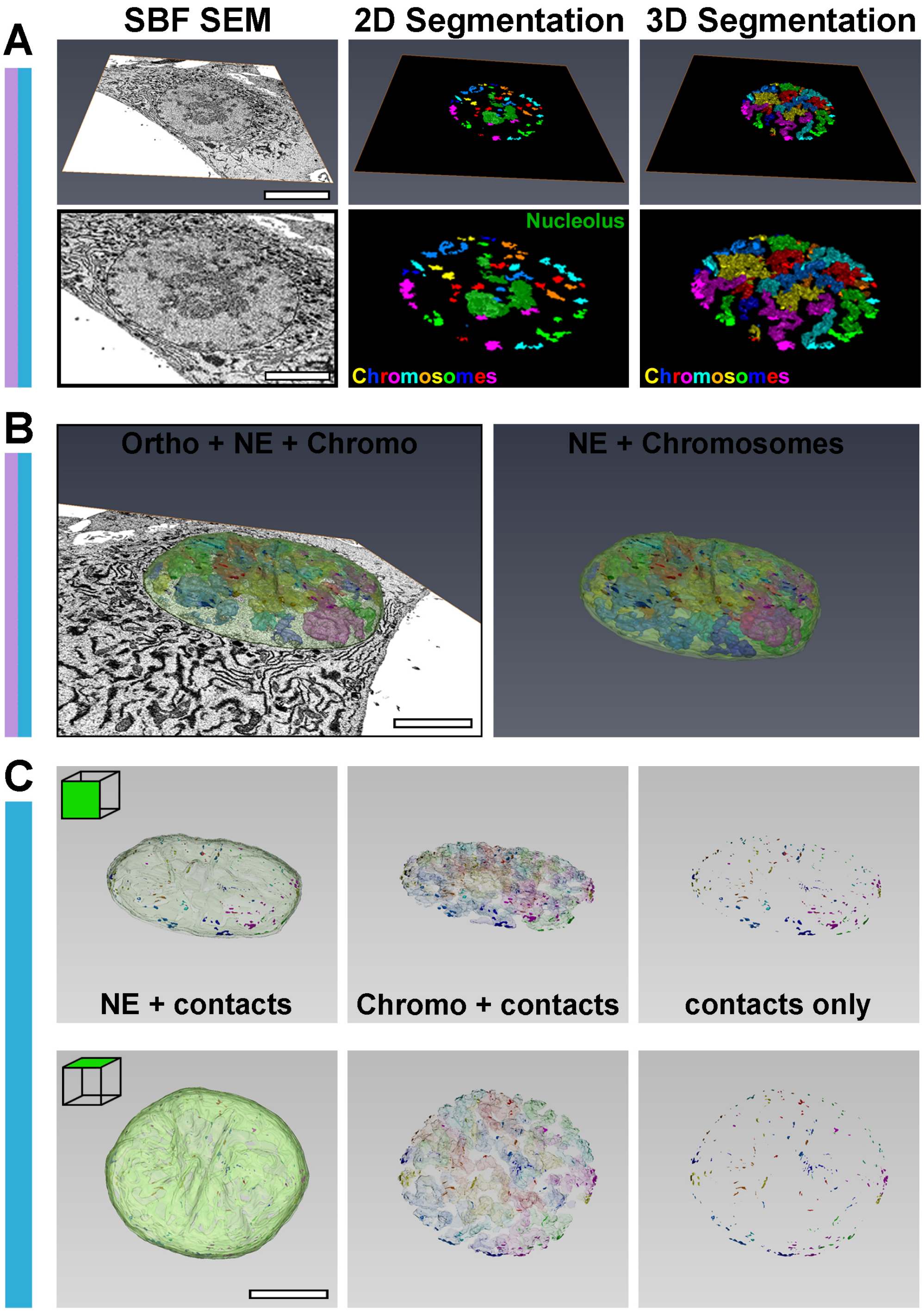

Supplementary Fig. 3: Almost all prophase chromosomes make contact with the NE. A) Representative cross section of a prophase cell. Visualised as EM orthoslice (left panels), 2D segmented chromosome orthoslice (middle panels), segmented chromosomes 2D ortholice plus 3D chromosome model. Bottom panel shows 2X zoom of the top panel. **B)** Reconstruction of the nuclear envelope (NE), shown with and without orthoslice. **C)** Digital footprint of chromosome/NE contact points. Data shown as NE (translucent green) plus contact points (left panels), contact points plus associated chromosome body (central panels), contact points alone (right panels). Chromosome/NE contact points have been left opaque in all images. Bars: A top – 10 µm, bottom – 5 µm; B and C – 5 µm.

Supplementary Figure 4 (related to Figures 3, 4 and 6)

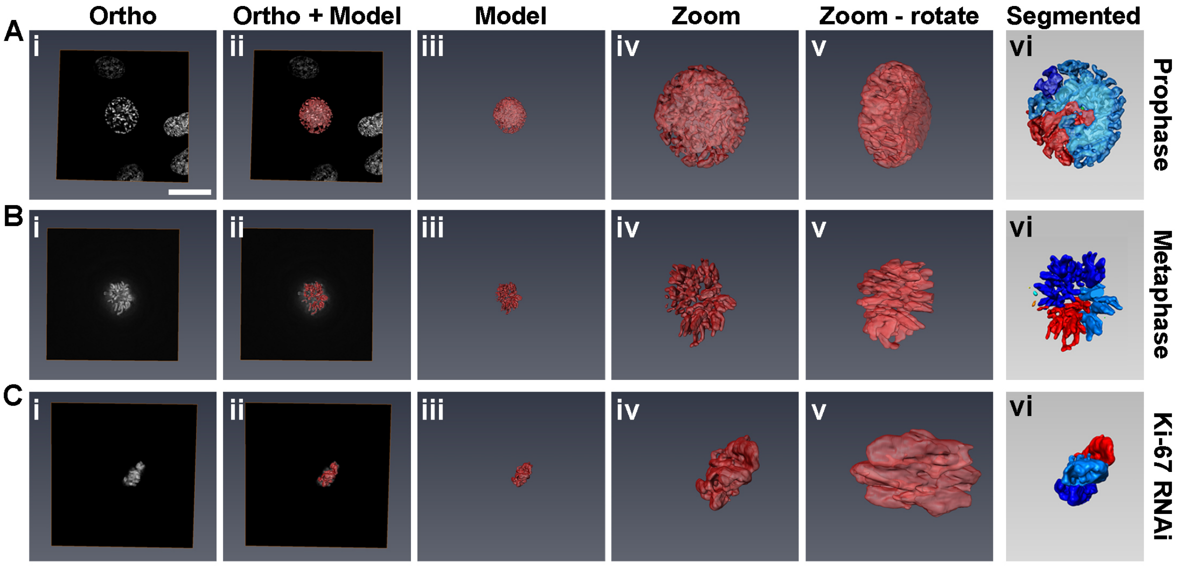

D

		Prophase	Metaphase	Ki67 RNAi
LIGHT MICROSCOPE	Volume (μm^3)	635	256	352
	Surface Area (μm^2)	1970	1196	585
	Chromosomes Segmented	8/46	14/46	4/46
ELECTRON MICROSCOPE	Volume (μm^3)	109.8	175.96	170.23
	Surface Area (μm^2)	1175.73	899.3	986.42
	Chromosomes Segmented	43/46	46/46	20/46

Supplementary Fig. 4: Direct comparison of correlative 3D modeling using DAPI and SBF-SEM reveals large discrepancies. Deconvolved DAPI signals from prophase (A), metaphase (B) and Ki-67 depleted cells (C), were modeled using Amira. Images show orthoslice alone (i), orthoslice plus model (ii) and model alone (iii-v). Segmentation tools, with identical parameters of their SBF-SEM counterparts, were attempted (vi). D) Summary table containing measurements from prophase, metaphase and Ki-67 depleted cells obtained from both light and electron microscopy informed data.

Supplementary Figure 5 (related to Figures 3 and 4)

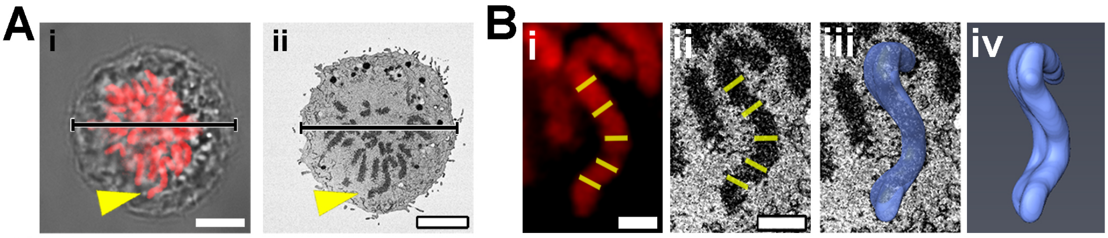

C

CELL		CHROMOSOME	
Light	EM	DAPI	EM
19.57 µm ± 1.03	18.66 µm ± 0.05	0.70 µm ± 0.07	0.69 µm ± 0.01

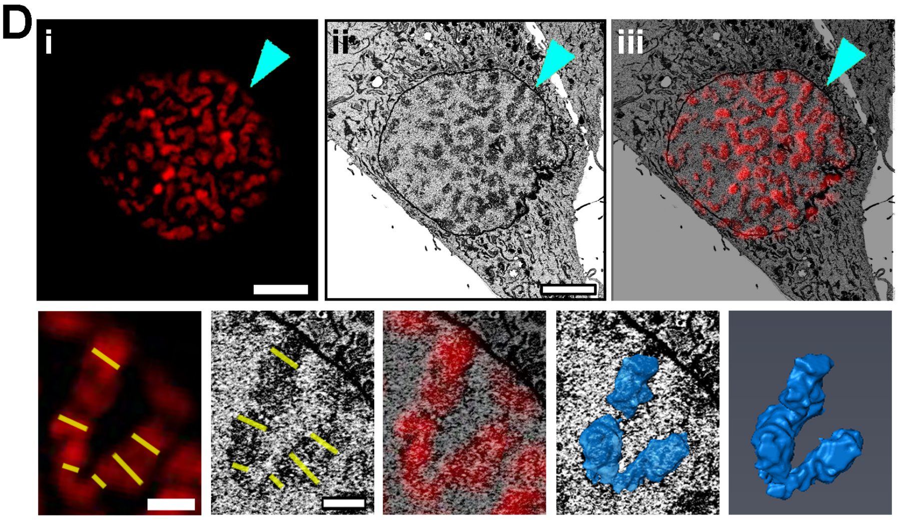

E

CHROMOSOME	
DAPI	EM
0.525 µm ± 0.124	0.528 µm ± 0.129

Supplementary Fig. 5: Direct comparison of 2D data acquired using DAPI and SBF-SEM are identical. Metaphase analysis (A-C). A) Whole cell comparison of metaphase. Metaphase cell diameter was measured using LM and SBF-SEM images. B) Zoom of chromosome from A (yellow arrow). Chromosome diameter was measured from correlative light (i) and SBF-SEM images (ii). The SBF-SEM informed model is also shown (iii-iv). C) Summary table of measurements. Prophase analysis (D-E). D) Correlative optical (i) and physical (ii) sections were used to measure prophase chromosome diameter. Overlay shown in iii. Zoom of chromosome from Di-iii (cyan arrow), represent an example of how chromosomes were measured using correlative light and SBF-SEM images. The SBF-SEM informed model is also shown (blue). E) Summary table of measurements. N = $6_{measurements}$ x $5_{chromosomes}$ for both prophase and metaphase cells. Bars: A, D = 5 µm, B, D (zoom) = 1 µm.

Supplementary Table 1 (related to Figures 4 and 5)

Segmented Units	Predicted Chromosome	Volume (um^3)	Surface Area (um^2)	DNA content (MB)	Compaction (MB per um^3 of chromosome envelope)	Length (um)	Width (um)	Width StDev (um)	Distance from Cell Center (um)	Volume W/O chromosome periphery (um^3)	Compaction (MB per um^3 of chromosome W/O periphery)
1	1	7.09	40.65	498	70.240	8.76	1.14	0.18	5.71	4.89	101.80
2	1	7.07	37.21	498	70.432	8.27	1.00	0.10	4.65	4.88	102.07
3	2	6.85	33.75	484	70.659	8.15	1.14	0.14	6.08	4.73	102.40
4	2	6.84	33.52	484	70.805	7.89	1.16	0.17	6.38	4.72	102.62
5	3	6.39	34.34	396	61.950	7.07	1.25	0.17	6.78	4.41	89.78
6	3	6.34	33.32	396	62.444	7.36	1.29	0.11	6.41	4.38	90.50
7	4	6.28	29.88	380	60.479	7.83	1.19	0.18	6.46	4.34	87.65
8	4	6.21	29.33	380	61.223	6.40	1.16	0.17	5.31	4.28	88.73
9	5	5.70	26.78	364	63.895	6.54	1.12	0.13	7.12	3.93	92.60
10	5	5.50	26.62	364	66.142	6.70	1.19	0.17	7.23	3.80	95.86
39	20	2.13	11.53	128	60.004	3.65	1.01	0.09	3.65	1.47	86.96
40	20	2.01	11.84	128	63.556	3.18	1.21	0.08	3.30	1.39	92.11
41	19	1.76	10.17	118	67.011	3.29	1.06	0.10	3.71	1.22	97.12
42	19	1.73	10.13	118	68.245	3.17	1.15	0.09	3.72	1.19	98.91
43	22	1.51	9.38	102	67.757	2.28	1.21	0.08	1.41	1.04	98.20
44	22	1.50	9.60	102	67.932	1.98	1.16	0.08	3.14	1.04	98.45
45	21	1.26	8.06	94	74.584	2.14	1.15	0.08	2.63	0.87	108.09
46	21	1.21	8.09	94	77.786	1.96	1.13	0.09	3.51	0.83	112.73
**Units 1-10, 39-46**	**SUM**	**77.4 um^3**	**404.2 um^2**	**5128 MB**	**Average of 66.95 Mb per um^3**	**NA**	**1.15 um**	**0.12 um**	**NA**	**53.39**	**Average of 97.03 Mb per um3**
**Units 1 - 46**	**COLLECTIVE SUM**	**175.96 um^3**	**899.3 um^2**	**12344 MB**	**Average of 70.15 Mb per um^3**	**NA**	**NA**	**NA**	**NA**	**121.46**	**Average of 101.55 Mb per um^3**

Supplementary Table 1: Summary table showing chromosome geometry measurements for characterised metaphase chromosomes, and collective summary from all 46 chromosomes (bottom row).

Supplementary Video – Figure Legends

Supplementary Video 1: Video of an alphoid^{tetO} HAC cell showing EM orthoslice (grey scale), modeled chromosomes (blue) and the HAC (green). Suppl. Video 1 related to Figure 2.

Supplementary Video 2: Video of an RPE1-hTERT cell in prophase showing EM orthoslice (grey scale), modeled chromosomes (red), and segmented chromosomes (multi-coloured). NE is shown in green. Digital footprint of chromosome to NE contacts is also shown. Suppl. Video 2 related to Figure 3.

Supplementary Video 3: Video of an RPE1-hTERT cell in metaphase showing EM orthoslice (grey scale), modeled chromosomes (red), and segmented chromosomes (multi-coloured). Suppl. Video 3 related to Figure 4.

SUPPLEMENTARY PROCEEDURES

Microscopy of Metaphase Spreads

Cells were treated with 0.2 µg/ml of colcemid for 3 hours before harvesting. Mitotic chromosomes were collected by mitotic shake off and cytospun onto poly-L-lysine coated slides. Samples were immersed in KCM buffer (10 mM Tris pH=8, 120 mM KCl, 20 mM NaCl, 0.5 mM EDTA, 0.1% Triton X-100) and IF staining was performed using KCM buffer with 1%BSA (Invitrogen). Afterwards, cells were fixed with 4% formaldehyde in KCM buffer and DNA was counterstained with DAPI. The following antibodies were used: rabbit anti-CENP-C (R555; 1/500), rabbit anti-SMC2 (1/1000), rabbit anti-Ki-67 (BD Transduction Lab 1/100 (Booth et al., 2014)), human anti-ACA (Earnshaw and Rothfield, 1985) (1/200). Images were acquired using a cooled CCD camera (CH350; Photometrics) on a wide-field microscope (DeltaVision Spectris; Applied Precision) with an 100X NA 1.4 Plan Apochromat lens. The data sets were deconvolved with softWoRx (Applied Precision).

Mass Spectrometry analysis

Correlation analysis data was mined from our proteomics archives (Ohta et al., 2010, Samejima et al., 2015). Protein-pairs with a strong correlation coefficient ($R \geq 0.80$) were extracted and used to draw the network diagram by Cytoscape.
Composite of chromosomes and breakdown of peripheral fractions.
The mass of proteins were deduced from copy number and their molecular weight.
Copy number of each protein on DT40 mitotic chromosomes was inferred by intensity based absolute quantification (iBAQ) algorithm. Spectral intensities of MS1 peak were analysed by MaxQuant 1.5.1.2 (Schwanhausser et al., 2011).

Calculations for chromosome geometry referred to within main text:

-1- A priori prediction of thickness of chromosome periphery in mitotic chromosomes.
Consider chromosome 1
Volume = 7.09 μm^3
diameter = 1.14 μm (radius = 0.57 μm)

Calculate the length (h) of chromosome 1, modeling the chromosome as a cylinder:
volume of a cylinder: $V = \pi \times r^2 \times h$
$7.09\ \mu m^3 = \pi \times (0.57\ \mu m)^2 \times h$
$h = 7/(\pi \times 0.3249) = 6.86\ \mu m$

Estimate the DNA volume in chromosome 1:
Total volume of metaphase chromosomes = 175 μm^3
Assumption: volume of prophase chromosomes (110 μm^3) = volume of the chromatin, as the nucleolus has not yet disassembled.
Periphery volume = 175 - 110 μm^3 = 65 μm^3
Fraction of chromosome volume that is chromatin = 110/(110 + 65) = 0.62857
Volume of chromatin in chromosome 1 = 0.62857 x 7.09 μm^3 = 4.457 μm^3

Diameter of chromatin cylinder in chromosome 1:

$V = \pi \times r^2 \times h$

so $4.457\ \mu m^3 = \pi \times r^2 \times 6.86$

$r^2 = 4.457/(\pi \times 6.86) = 0.207$

$r = 0.455$

Diameter of the chromatin = 2r = 0.91

Thickness of periphery of chromosome 1 =

(chromosome diameter – chromatin diameter)/2

= (1.14 - 0.91)/2 = 115 nm

This "first principles" calculation of the periphery thickness gives a value dead in the middle of our observed range of 87 - 150 nm.

However:

-1- This calculation assumes that the compaction of the chromatin is the same in prophase and mitosis. It also ignores the actual thickness of the chromosome periphery measured on mitotic chromosomes.

Measurements of the thickness of the metaphase periphery by EM:

Booth (Booth et al., 2014) ~150 nm

Hernandez-Verdun and co-workers ~ 143 nm (measured by us from their published micrographs (Gautier et al., 1992a).

Assume a thickness of the periphery at metaphase of 150 nm.

If the diameter of chromosome 1 is: 1.14 μm

The diameter of the chromatin cylinder = 1.14 - (2 x 0.15) μm = 0.84 μm

This gives r = 0.42 μm —> this is the radius of the chromatin in chromosome 1

$V_{chromatin} = \pi \times r^2 \times h = \pi \times (0.42\ \mu m)^2 \times 6.86\ \mu m = 3.80\ \mu m^3$

The fraction of the volume of chromosome 1 that is chromatin = 3.80/7.09 = 0.536

If the thickness of the periphery is 150 nm, then 54% of the total metaphase chromosome volume is chromatin.

-3- How much do chromosomes compact between prophase and metaphase?

The total volume of metaphase mitotic chromosomes is 175 μm^3.

0.54 x 175 μm^3 = 93.8 μm^3.

This means that between the prophase cell that we imaged and the metaphase cell that we imaged, the chromatin volume compacted by (110/93.8)-1 x 100 = 17%

-4- Calculation of the predicted periphery thickness in Ki-67-depleted chromosomes:

Total volume of the chromatin in metaphase chromosomes estimated from light microscopy: 256 μm^3.

Total volume of the chromatin in Ki-67-depleted metaphase chromosomes estimated from light microscopy: 352 μm^3.

Ratio of Ki-67/wild-type volume: 1.375

Metaphase chromosome 1 chromatin volume calculated above from EM: 3.80 μm^3.

Normalized volume of Ki-67-depleted metaphase chromosome 1 chromatin: 5.225 μm^3.

Measured total volume of metaphase chromosome 1: 7.09 μm^3.

Measured total volume of wild type chromosomes: 175 μm^3.

Measured total volume of Ki-67-depleted chromosomes: 170 μm^3.

Corrected volume of chromosome (170/175) x 7.09 = 6.89 μm^3.

Since the total volume is so similar, **<u>assume</u>** that the linear measurements (e.g. radius and length) of Ki-67-depleted chromosomes will be similar to wild type:

$V_{chromatin} = \pi \times r^2 \times h$

$5.225\ \mu m^3 = \pi \times r^2 \times 6.86\ \mu m$

$5.225/(\pi \times 6.86)\ \mu m^2 = r^2$

$.4925\ \mu m = r$

$D = 0.985\ \mu m$

Likely thickness of periphery compartment in Ki-67-depleted chromosome 1:

$(1.14 - 0.985)/2 = 77\ \mu m$

Percentage of chromosome 1 that is chromatin = $5.225\ \mu m^3 / 6.89\ \mu m^3 = 76\%$
